# Global Color Variation in Giant Hornet *Vespa mandarinia* Smith (Hymenoptera: Vespidae) and Potential Threat of Spread of the Commercially Reared “Hongniang” Phenotype

**DOI:** 10.3390/insects17090934

**Published:** 2026-09-07

**Authors:** Xiao-Juan Huang, Jia Li, Huan-Yi Guo, Yi Ran, Jia-Qi Kang, Yao Zhang, Tong Zhou, Chen-Lu Yang, Yi-Cen He, Si-Yuan Yue, Yuan Hua, Cornelis van Achterberg, Li-Jun Cai, Jiang-Li Tan

**Affiliations:** 1 Shaanxi Key Laboratory for Animal Conservation/Key Laboratory of Resource Biology and Biotechnology in Western China, College of Life Sciences, Northwest University, Xi’an 710069, China; 2 Division of Bioscience, University College London, Gower Street, London WC1E6BT, UK; 3 Naturalis Biodiversity Center, 2333 CR Leiden, The Netherlands; 4 State Key Laboratory of Agricultural and Forestry Biosecurity, College of Plant Protection, Fujian Agriculture and Forestry University, Fuzhou 350002, China

**Keywords:** *Vespa mandarinia*, killer wasps, polymorphism, dispersion, anthropogenic invasion

## Abstract

The largest giant hornet *Vespa mandarinia* (VM) as a notorious “murder hornet” exhibits a complex color polymorphism and has been reared and directly released into the wild for commercial purposes in China, raising concerns that it may become an anthropogenic invasion disaster. Compiling the collections, as well as the global data online, a comprehensive description, modeling, and analysis on color variations and distribution patterns were conducted. A total of 42 images are presented firstly to represent all identified color phenotypes, which are categorized into four main color varieties, including a new variety, *Vespa mandarinia* var. *pseudomagnifica* (VMP). Prediction models show that VM is mainly native in Asia; the “400 mm isohyet” in China is a major biogeographic barrier limiting the northwestward expansion. Over the past decade, the distribution area of *Vespa mandarinia* var. *magnifica* (VMF) has probably expanded significantly (from 121.25 × 10^4^ km^2^ to 260.29 × 10^4^ km^2^), particularly in China. The potential distribution of VMF and *Vespa mandarinia* var. *mandarinia* (VMM) shaped the typical asymmetry of biogeographic pattern and highlight the west Palaearctic–Oriental boundary. These findings provide a scientific basis for risk assessment, inform monitoring, and management decisions related to the commercial rearing and population control of expanding hornet populations.

## Simple Summary

The giant hornet, *Vespa mandarinia* (VM), is well known for being the largest social wasp and potentially having the strongest aggressive attacking capability. However, over the past decade, it has been artificially bred and released indiscriminately into the wild for commercial use in China. This study presents the first global systematic analysis of the color polymorphism and distribution pattern of VM. The results indicate that VM is primarily native to Asia with the largest suitable habitat concentrated in China. The “400 mm isohyet” in China acts as a major biogeographic barrier limiting the northwestward range expansion. The east edges of Western Sichuan Plateau and Hengduan Mountains act as a para-“Wallace Line” within China, which shapes the biogeographic distribution of *Vespa mandarinia* var. *magnifica* (VMF) and *Vespa mandarinia* var. *mandarinia* (VMM), forming a distinct asymmetrical biogeographic pattern that highlights the west Palaearctic–Oriental boundary. These findings not only provide a new animal model and biological evidence for resolving the academic debate regarding the biogeographic demarcation between the Palaearctic and Oriental realms, but they also offer a scientific foundation for the effective population management of hornets.

## 1. Introduction

Color polymorphism, in which multiple discrete color phenotypes occur within a single species, has been extensively documented in various animals especially insects [1,2,3,4,5]. Traditionally, color polymorphism has been regarded as an evolutionary adaptation enabling species to occupy a wider range of habitats or niches [6]. A series of hypotheses have been proposed to explain potential functions of color variations in diverse habitats, including camouflage, thermoregulation, mechanical resistance, and UV protection [7,8]. Understanding the factors shaping the formation of these specific distribution areas is essential for comprehending the evolutionary processes, ecological adaptation, and biodiversity conservation.

The giant hornet *Vespa mandarinia* (VM) Smith (Hymenoptera: Vespidae), described from Ningbo (Zhejiang, China) in 1852, is native to subtropical and temperate mountainous regions of Asia and exhibits pronounced color polymorphism, with majority of the color variations observed in China. Previous authors have named eight subspecies based solely on color patterns: viz. *Vespa mandarinia mandarinia* Smith, 1852 [9], *Vespa mandarinia nobilis* Sonan, 1929 [10], *Vespa mandarinia magnifica* Smith, 1852 [11], *Vespa mandarinia magnifica* var. *bellona* Smith, 1871 [12], *Vespa mandarinia latilineata* Cameron, 1903 [13], *Vespa mandarinia japonica* Radoszkowski, 1857 [14], *Vespa mandarinia soror* Buysson, 1905 [15], and *Vespa magnifica sonani* Matsumura, 1930 [16]. Archer (1995) [17] upgraded *Vespa mandarinia soror* into a valid species, *Vespa soror* Buysson, and reduced the number of subspecies into three color forms: viz. “*mandarinia*” (VMM, Eastern China, Japan), “*magnifica*” (VMF, Northeastern India to Southwestern China), and “*nobilis*” (VMN, Taiwan of China). However, the paucity of specimens in their collections limited the understanding of their distribution. Although all subspecies were synonymized by Carpenter & Kojima (1997) [18], people still habitually refer to the color forms as var. or subspecies, even species. Some researchers still confuse the invalid name var. *japonica* with VMM [19].

Among the various color variants, one variety (-*bellona*) of *V. mandarinia* var. *magnifica* (VMF-b), commonly named “Hongniang”, originally occurring in the adjacent area of Southwestern China (mainly in Yunnan) and Mainland Southeast Asia, has been successfully bred for its delicious taste and possible medicinal value, resulting in a rapid spread into 14 provinces of China since 2014 [20]. It should be emphasized that this kind of breeding behavior is not a closed process. Instead, large-scale breeders only cultivate overwintering queens until the initial nest is built in the following spring and then sell them to other retail investors who release the colony into the wild without authorization. This rapid trans-regional introduction and spread have created both practical management challenges and fundamental ecological questions about the environmental adaptability of this morph. For example, what is its potentially suitable habitat, and what is its dispersal risk outside its native range? Additionally, the color polymorphism in *V. mandarinia* (VM) provides an excellent model for addressing a classic evolutionary question: in a context of potential geographical isolation, is its color differentiation primarily driven by neutral processes such as genetic drift, or is it shaped by adaptive selection in response to heterogeneous environmental conditions?

The Maximum Entropy (MaxEnt) model, benefiting from its prominent advantage of achieving high-precision modeling with limited small samples, has been applied in multiple research fields, such as species-suitable habitat distribution modeling, invasive species early warning, assessment of the impacts of climate change on organismal distribution, prediction of agricultural pests and diseases, etc. [21,22,23]. These models have demonstrated strong application potential in Hymenoptera. For instance, the models have successfully applied to predict the distribution of *Vespa velutina* [24,25].

Herein, we illustrate the color variations of *V. mandarinia* (VM), describe the distribution pattern of color phenotypes of VM, and analyze the potential constraints of climatic factors (e.g., precipitation and temperature) on phenotypic differentiation. Additionally, we integrate the distribution data of VM (with all color phenotypes), respectively, using distribution modelling and spatial analysis to predict their potential distribution. The research aims to reveal the geographically structured patterns of species phenotypic differentiation driven by climate and to assess ecological risks, thereby offering essential scientific insights for biodiversity conservation and invasive species management.

## 2. Materials and Methods

### 2.1. Color Pattern Analyses

**Figure 1:**
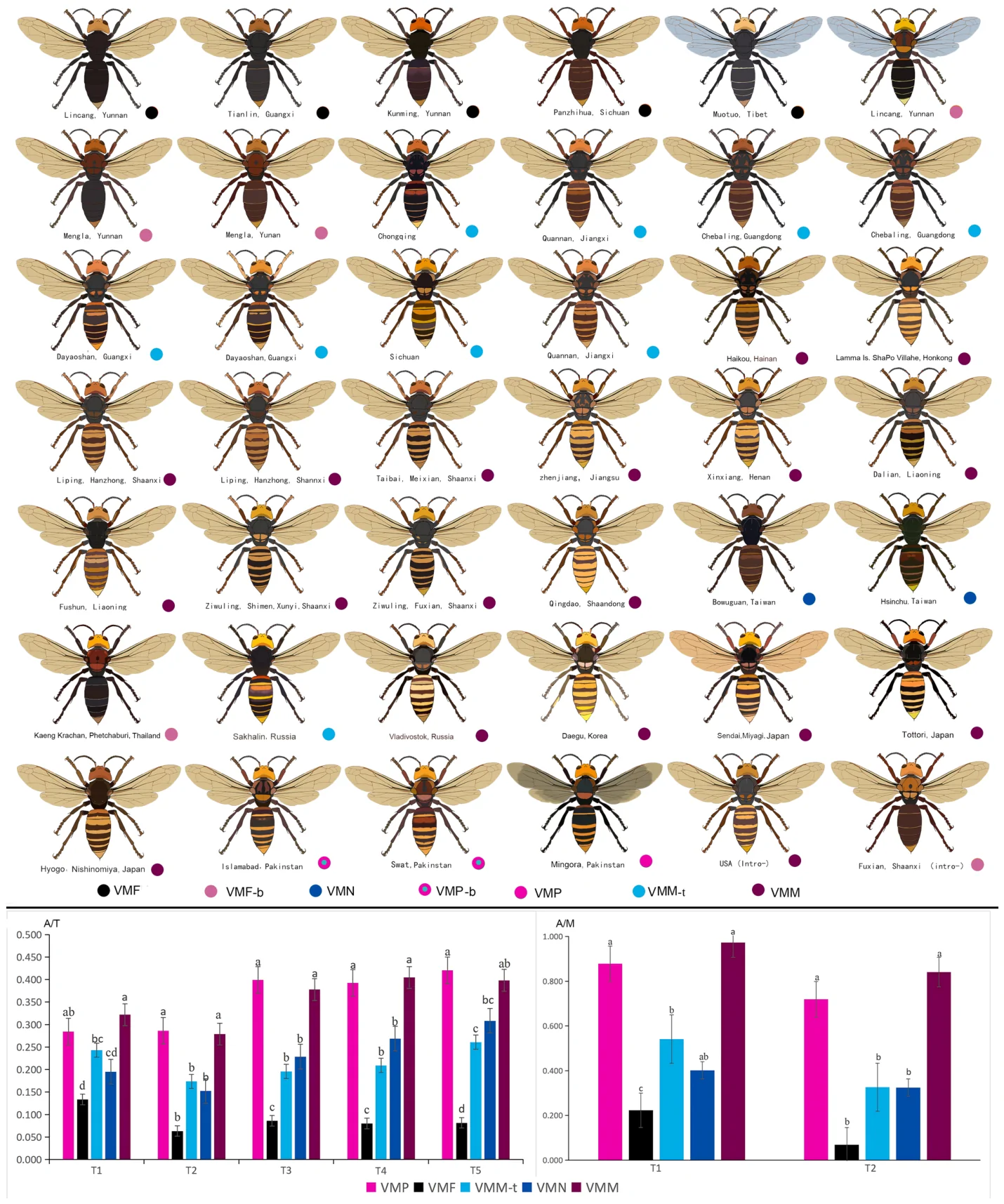
Color phenotypes of *Vespa mandarinia* (**upper**), bar chart of the average ratio of the width of each yellow-colored apical band (A1–5) to the length of the corresponding tergum (T1–5) (**lower left**), and the ratios of the width of each yellow-colored apical band (A1–2) to the width of the dark-colored mid-band (M1–2) (**lower right**). Abbr.: VMF, *Vespa mandarinia* var. *magnifica*; VMF-b, *Vespa mandarinia* var. *magnifica*-*bellona*; VMN, *Vespa mandarinia* var. *nobilis*; VMP, *Vespa mandarinia* var. *pseudomagnifica*; VMP-b, *Vespa mandarinia* var. *pseudomagnifica*-*bellona*; VMM, *Vespa mandarinia* var. *mandarinia*; VMM-t, transitional phenotypes between VMF and VMM. Different letters above the columns indicate significant differences (*p* < 0.05).

The identification of color forms was mainly based on Archer (1995, 2012) and van der Vecht (1959) [17,26,27]. More than 300 specimens (in collections of NWUX (Xi’an), RMNH (Leiden), MNHN (Paris) and TARI (Taichung)) and more than 2000 pictures (with location data) on “iNaturalist” were checked. A total of 42 pictures were illustrated, representing all color phenotypes (Figure 1 upper). We painted color onto our line drawing draft model by Adobe Photoshop 2024, following the specimen photographs. Considering that the observation of a specimen’s natural color is limited by its three-dimensional structure and preservation condition, we did not take into account variations in color saturation or vividness for entries categorized under the same color form. In the same colony, individuals could vary with light color patches on the anterior part of terga 1–2 (T1–2), such as specimens of Liping, Hanzhong, Shaanxi, etc., in Figure 1; large or small ferruginous patches on the mesosoma are often observed. However, only stable color pattern is the primary diagnostic trait for identifying subspecies/varieties. Therefore, we not only followed the framework of Archer [17,26], which focuses on the relative width of the bands on T1–5 for geographic variety analysis, but also proposed our own opinion on VMN to clarify the materials of VMM-t (with transitional characters between VMN and VMM).

### 2.2. Species Distribution Data

**Figure 2:**
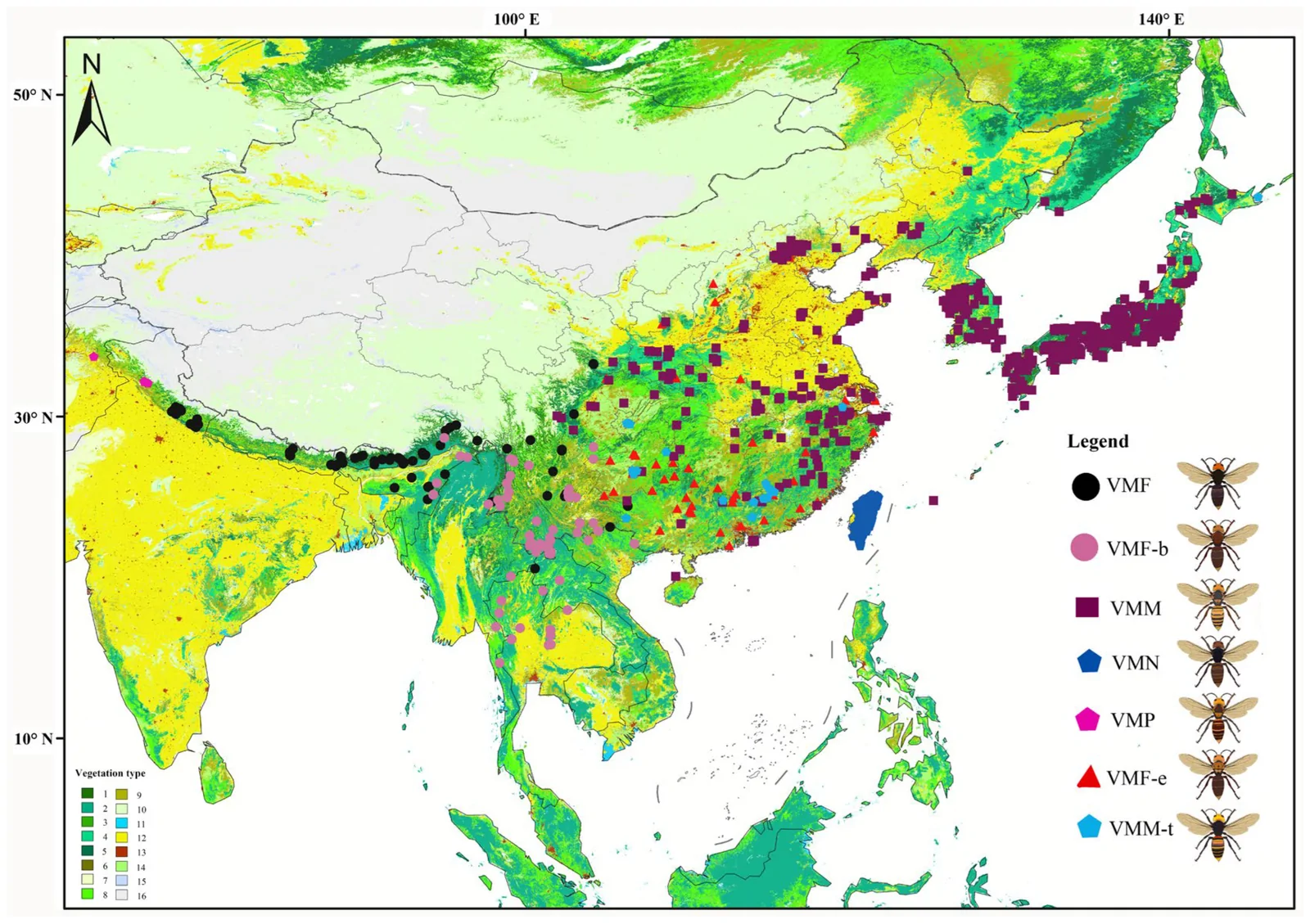
Map of distribution of the main color morphs in *Vespa mandarinia* (VM). Abbr.: VMF, *Vespa mandarinia* var. *magnifica*; VMF-b, *Vespa mandarinia* var. *magnifica*-*bellona*; VMM, *Vespa mandarina* var. *mandarinia*; VMN, *Vespa mandarinia* var. *nobilis*; VMP, *Vespa mandarinia* var. *pseudomagnifica*; VMF-e, *Vespa mandarinia*. var. *magnifica* expansion (possibly due to rearing by people, its new distribution record in China after 2014 was supposed to be expansion, according to Huang et al. (2018) [20]). Vegetation Type: 1 Evergreen Needleleaf Forest; 2 Evergreen Broadleaf Forest; 3 Deciduous Needleleaf Forest; 4 Deciduous Broadleaf Forest; 5 Mixed Forest; 6 Closed Shrubland; 7 Open Shrubland; 8 Woody Savanna; 9 Savanna; 10 Grassland; 11 Permanent Wetland; 12 Cropland; 13 Urban and Built-up Land; 14 Cropland/Natural Vegetation Mosaic; 15 Snow and Ice;16 Barren or Sparsely Vegetated.

The data of this species were assembled by our research team over 15 years of collecting specimens of Vespidae from China and checking collection in the museums combined with online data (from iNaturalist (https://www.inaturalist.org, accessed on 20 March 2026)). A total of 2165 distribution records (including 257 VMF, 307 VMN, 1572 VMM, 23 VMM-t, and 6 VMP, as well as a new variety, *Vespa mandarinia* var. *pseudomagnifica*) were shown (Figure 2). ArcGIS v10.8 was used to perform spatial thinning analysis to decrease the impact of model over-fitting resulting from the over-closed (smaller than 5 km) distribution records. After preprocessing, the MaxEnt modeling in this study included 185 occurrence records for VMF, 149 for VMN, and 734 for VMM.

### 2.3. Climate Variables and Data Processing

The 19 climate factors, representing the average values for the period from 1970–2000, were selected and sourced from the WorldClim (http://www.worldclim.org/, accessed on 19 October, 2025) for the MaxEnt model. An initial exploratory model was generated. As a result, we obtained the percentage contribution and permutation importance as measures of variable importance (Table S1). The Spearman correlation between pairs of variables was calculated (Table S2). To avoid potential over-fitting and multicollinearity, variables were first ranked by their percentage contribution to the exploratory model. Starting from the highest-contribution variable, we sequentially retained variables, and when a pair of variables showed a Spearman’s correlation coefficient ≥ 0.8, the one with the lower contribution was excluded. Seven key variables (Bio1 Annual mean temperature, Bio3 Isothermality, Bio4 Temperature seasonality (standard deviation × 100), Bio8 Mean temperature of wettest quarter, Bio14 Precipitation of driest month, Bio15 Precipitation seasonality, and Bio18 Precipitation of warmest quarter) were finally selected. Then we reconstructed the distribution model using the seven key climate variables. For future climate data, we selected three scenarios from the new generation climate system model BCC-CSM2-MR developed by the Beijing Climate Center (BCC), SSP126 (low greenhouse gas emission scenario), SSP245 (medium greenhouse gas emission scenario), and SSP585 (high greenhouse gas emission scenario), to predict the distribution of this species.

### 2.4. Model Analysis and Parameter Configuration

To explore the impact of environmental factors on the potential distribution of each subspecies, we separately performed MaxEnt modeling for VMF, VMN, and VMM using their respective occurrence records. Owing to insufficient available specimens, and the fact that VMM-t does not constitute an independent color morph, VMM-t and VMP were excluded from the MaxEnt analyses. MaxEnt 3.4.4 software was used to simulate its potential distribution. We used the software’s default setting of 10,000 background points, which were randomly generated within the global landmass. To reduce uncertainty caused by the random selection of data, 75% of the distribution data were randomly selected for training, while the remaining 25% were used as the testing dataset, and the replicated run type was set as “Subsample”. The training process was repeated 100 times, with other parameters set to their default values [28]. Jackknife analysis was used to determine the contribution rates of key environmental variables and then derived the logistic relationship between distribution probability and climate factors through response curves [29].

### 2.5. Model Evaluation

The Receiver Operating Characteristic (ROC) curve is used to assess the accuracy of model predictions, with the Area Under the Curve (AUC) under the ROC curve serving as a performance indicator for MaxEnt predictions [30]. The AUC ranges from 0.5 to 1, with values closer to 1 indicating higher model accuracy [31]. An AUC value of 0.5–0.6 indicates failed performance; 0.6–0.7 indicates poor performance; 0.8–0.9 indicates excellent results; and values above 0.9 indicate high performance.

### 2.6. Suitable Habitat Classification

Based on the Intergovernmental Panel on Climate Change (IPCC) likelihood report divisions for assessment methods [32], combined with the actual situation of VMF, its habitat suitability is classified into four levels and represented in different colours: unsuitable areas (*p* < 0.1, white), low suitability areas (0.1 ≤ *p* < 0.3, yellow), moderate suitability areas (0.3 ≤ *p* < 0.5, orange), and high suitability areas (0.5 ≤ *p*, red). Among them, the presence/absence boundary was determined using the Maximum Training Sensitivity plus Specificity (MTSS) criterion [33]. Based on the average MTSS values derived from csv file across all replicate runs, a threshold of approximately 0.1 was adopted to distinguish unsuitable from suitable habitats. To further subdivide suitable habitats for visualization and area statistics, we employed the Natural Breaks method in ArcGIS. Across the four subspecies, the optimal breakpoints averaged rounding to 0.3 (low-vs-moderate,) and rounding to 0.5 (moderate-vs-high). Using the SDM Toolbox 2.0, we calculated the centroid position of VMF in different periods to determine the migration direction of the distribution area of VMF while using the centroid distribution to evaluate the migration of its suitable range.

### 2.7. Multivariate Environmental Similarity Surface and Most Dissimilar Variable Analyses

The Multivariate Environmental Similarity Surface (MESS) was employed to assess the climatic similarity between each projection grid cell and a reference set of environmental variables. For each variable Vi in the reference layer, we defined its minimum as mini and maximum as maxi, with pi representing the value at a given point P. The percentage of reference points with values below pi was denoted as fi. When fi equals 0, the similarity score for that variable is computed as 100 × (pi − mini)/(maxi − mini). For fi values between 0 and 50, the score is 2fi; for fi between 50 and 100, it is 2(100 − fi); and when fi equals 100, the score is 100 × (maxi − pi)/(maxi − mini). The overall MESS value at point P is the minimum score across all environmental variables, also termed the “Most Dissimilar Variable” (MoD). Negative MESS values indicate that at least one variable exceeds the environmental range observed in the reference dataset, signaling a climatic anomaly. Such conditions imply that the projected climate at point P may fall outside the species’ ecological tolerance, thereby identifying areas of potential vulnerability under future climate change. A MESS value of 100 denotes complete climatic consistency with the reference conditions. These thresholds provide a useful framework for delineating the environmental limits of species’ habitats and for detecting regions potentially subject to ecological stress. All MESS and MoD calculations were performed using the density.tool.novel module implemented in MaxEnt version 3.4.4 [34,35].

## 3. Results

### 3.1. Color Polymorphism of Vespa mandarinia (VM) and Its Distribution Pattern

The color variations of the *V. mandarinia* (VM) are complex. After examining more than 300 specimens and 2000 online images, a total of 42 specimens were selected and illustrated to show the continuous color variation (Figure 1, upper). The ratios of the width of each yellow-colored apical band (A1–5) to the length of the corresponding tergum (T1–5) were measured and compared separately, as well as the ratios of the width of each yellow-colored apical band (A1–2) to the width of the dark-colored intermediate band (M1–2). The average measurement results are presented in a bar chart (Figure 1, lower). A total of 158 samples (including of 26 VMF, 12 VMN, 15 (from Japan) +79 (from China) +9 (from Korea) VMM, 13 VMM-t, and 4 VMP were selected and measured.

Combining the color characteristics with the relative isolation of geographical distributions, we tentatively divided the species into four basal color forms. (1) *Vespa mandarinia* var. *magnifica* (VMF), characterized as very narrow, was more or less restricted to apical rims of the terga 1–5 (T1–5). Normally there are two bands (a broad anterior dark-coloured band, very narrow posterior light-coloured band) available on T1–2; sometimes light-coloured patches appeared on anterior part of T1 (Figure 1). Among them, some high mountain specimens are almost entirely dark (the local-named “Dahei Hongniang” means totally black “Hongniang”). The mesosoma of common VMF was black (the local named “heibei Hongniang”), or with large ferruginous patches (the local name “Hongniang”, named “bellona” by researchers, VMF-b). Farmers prefer to select VMF, especially the variety VMF-b for breeding. Although it was previously confined to Mainland Southeast Asia and Southwestern China, it has spread widely across China (possibly through commercial channels) since 2014 [20]. The expansion sites were marked according to our survey and online data (Figure 2). (2) *V. mandarinia* var. *mandarinia* (VMM), generally characterized as T1–2 with three bands (basal and apical bands that are light coloured, an intermediate band that is dark coloured; three bands more or less of same width). Apical bands of T3–5 broad extend for at least one-third length of the corresponding tergum (Figure 1). VMM populations are widely distributed in China (ranging westward to the Western Sichuan Plateau and the eastern margin of the Hengduan Mountains, northward to Liaoning). The color form from Hong Kong and Hainan Island, which are located close to the Chinese mainland, also belong to the typical VMM. The specimens from Korea, Japan, and the Russian Far East all belong to VMM. Interestingly, specimens in the upper three countries have a black mesoscutum, while many specimens collected in China bear more or less small ferruginous patches (Figure 1). The results support the opinion of Archer (1995) [17] that var. *japonica* and var. *latilineata* both are synonyms of VMM. The specimens report from USA (Figure 1, USA-intro) fits well to VMM. Additionally, a few specimens exhibit relatively narrow apical margins, which are very similar to those of Taiwan, China. However, thinking of the specimens collected from the same location and date, which showed inter-individual variation in the width of apical bands of T1–5, indicated that they were possibly in the same nest. As a result, we temporarily classify these specimens as VMM but label them as VMM-t (Figure 1) to reflect their transitional nature. Interestingly, most VMM-t is distributed on the border between VMF and VMM, coincidentally in the northwestern direction of Taiwan Island. This distribution pattern may result from gene flow between these two color variants, with the population of Taiwan being isolated by the sea. (3) *V. mandarinia* var. *nobilis* (VMN), according to Archer (1995) [17], as an intermediate color forms between VMF and *V. mandarinia* var. *mandarinia* (VMM), characterized as T1–2 with three bands (an anterior light-coloured band that is not very distinct, an intermediate dark-coloured band, and a much narrower posterior light-coloured band); each band of T3–5 narrow extends less than one-third length of the corresponding tergum but is not restricted, more or less, to rims (Figure 1). Archer mentioned VMN was restricted to Taiwan Island [17,26]. However, no statistically meaningful difference exist between VMM-t and VMN (Figure 1, lower). We propose three extra characters of VMN as follows: (a) metasomal bands that are more or less reddish (yellow in VMM-t); (b) mesoscutellum that is completely black (completely black or with light patches in VMM-t); (c) basal band of T2 that are reddish, more or less extending backwards laterally. At most, there are two indistinct yellow spots on basal T2 (with a distinct anterior yellow band, which commonly does not extend backwards laterally in VMM-t). Only when the three characters are all satisfied could it be considered as a real VMN. (4) *V. mandarinia* var. *pseudomagnifica* (VMP) is a firstly reported variation, located in the northern mountains of Pakistan and Western India. Specimen data online indicated that it is similar to VMF with the apical band of T1, which is relatively narrow, and with the mesoscutum black completely or black with reddish brown patches (VMP-b). However, the apical bands of T2–5 are wide as that of VMM (Figure 1, lower).

According to the compiling data, the distribution map of the main color morphs of *Vespa mandarinia* (VM) is provided (Figure 2). It is clearly visible that the distribution of VM was restricted by the 400 mm precipitation line (“400 mm isohyet”) in China (the boundary between the semi-humid region (forest vegetation and crop land, note 12 in Figure 2) and semiarid climate (grassland vegetation, notes 10 in Figure 2) in China). Four main color forms are distributed in a continuous manner, with VMF tending to be more present in the southwest, subtropical area; VMM occurs in the northeast temperate zone, except some VMM-t occurs in between. VMN distributes in Taiwan, China. Although VMF was originally present in subtropical areas, it has extensively surpassed the west boundary of VMM (Figure 2). Interestingly, the mesonotum of VMF-b and VMP-b are both in ferruginous color mainly. However, VMF-b is distributed in humid subtropical areas, while VMP-b occurs in dry subtropical areas.

### 3.2. Model Performance and Key Environmental Variables for VM

The area under the curve (AUC) is a metric that reflects the accuracy of MaxEnt model simulations. In the study, the MaxEnt model achieved an average AUC of 0.902 ± 0.002 (Figure S1). The high level of accuracy underscores the model’s reliability and supports its use as a foundation for the current investigation. Using Pearson correlation coefficients and contribution rate, six key environmental variables were identified and incorporated into the species distribution model (Tables S1 and S2). Jackknife analysis revealed the contribution of each variable as follows: Bio18 (58.7%), Bio15 (10.8%), Bio3 (7.4%), Bio4 (5.8%), Bio1 (1%), and Bio8 (0.8%), with a total importance score of 100% (Table S1). These results indicate that the selected environmental variables effectively simulate the potential distribution of VMF.

### 3.3. The Potential Distribution of VMF, VMN, and VMM in the Current Period

**Figure 3:**
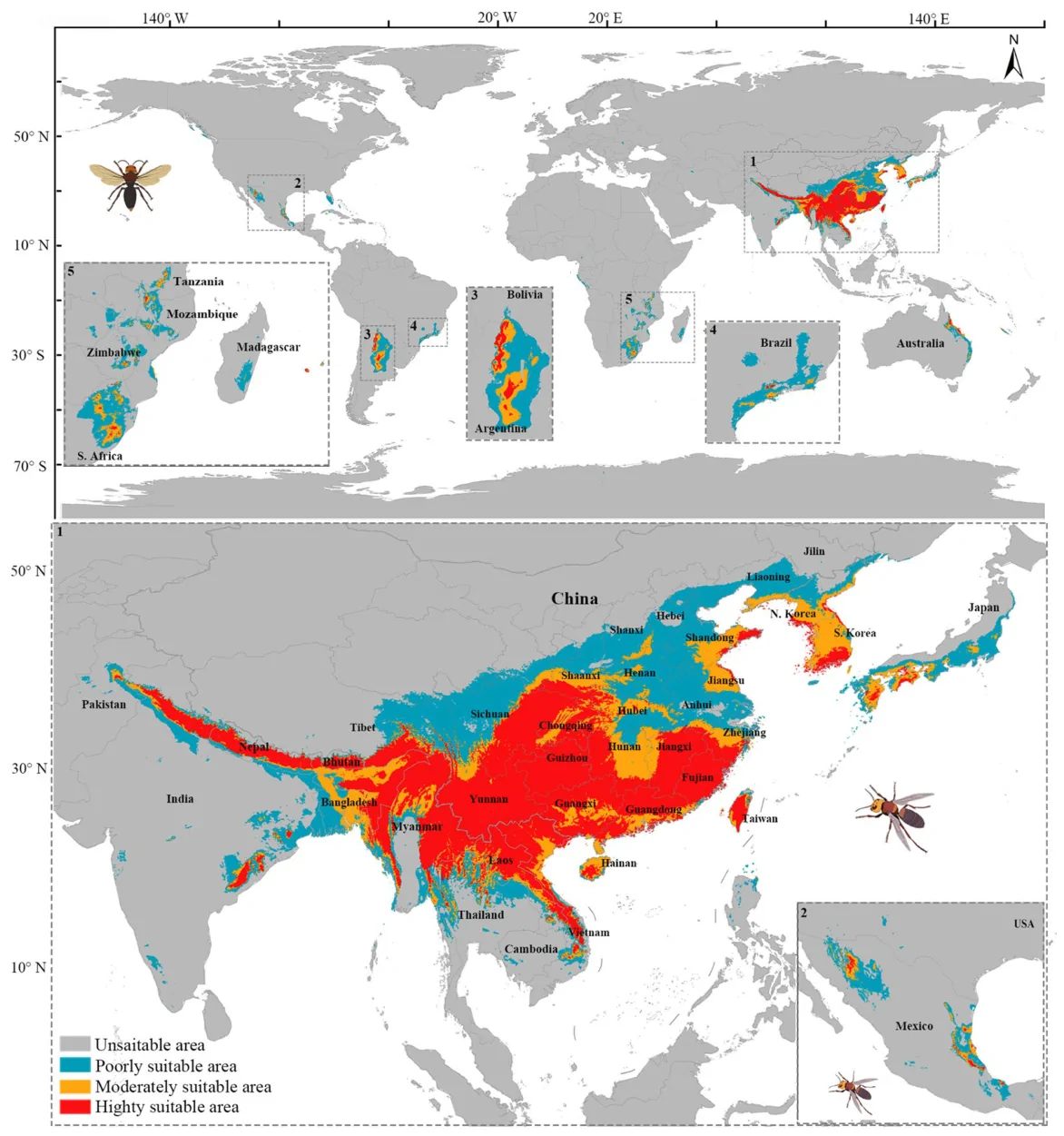
The suitable habitat distribution of VMF varies across different regions of world under the current climate conditions. Note: The total areas are categorized into four zones: high (red), moderate (orange), low (blue), and unsuitable (grey). Abbr.: VMF, *Vespa mandarinia* var. *magnifica*.

**Figure 4:**
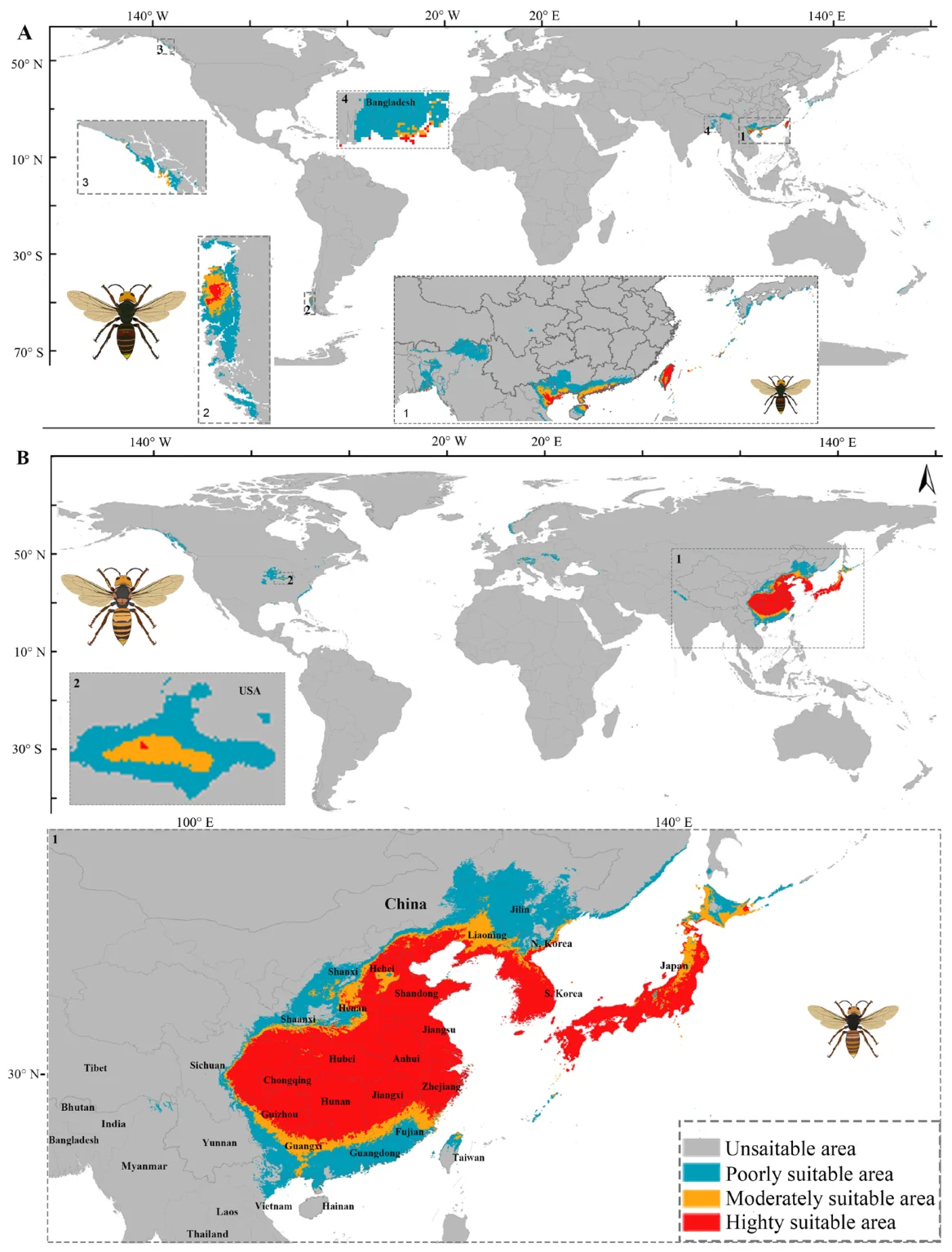
The suitable habitat distribution of VMN (**A**) and VMM (**B**) varies across different regions of world under the current climate conditions. Note: The total areas are categorized into four zones: high (red), moderate (orange), low (blue), and unsuitable (grey). Abbr.: VMM, *Vespa mandarinia* var. *mandarinia*; VMN, *Vespa mandarinia* var. *nobilis*.

**Figure 5:**
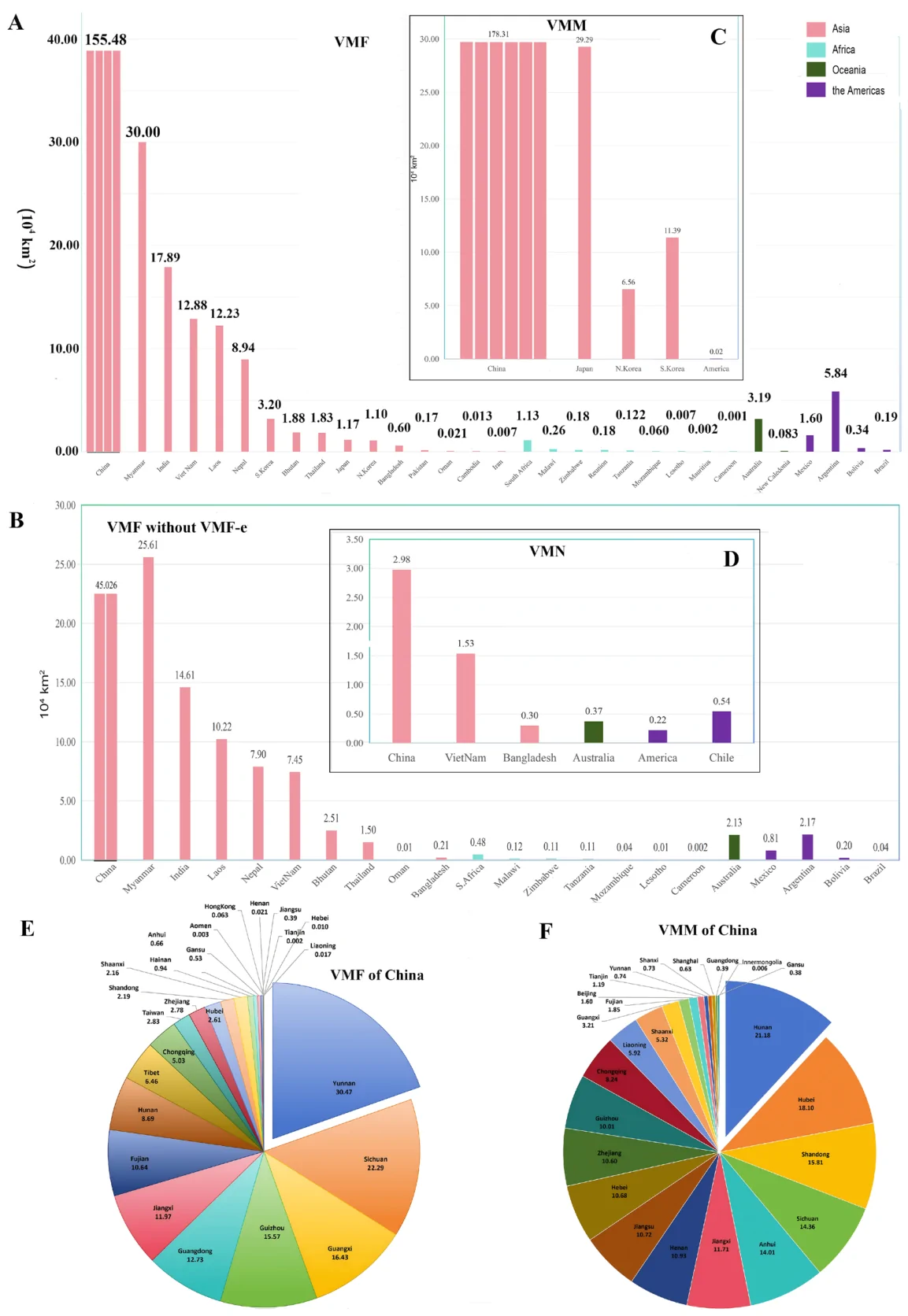
Comparison of predictions on the area of highly suitable habitats of the color forms of VM. (**A**–**D**). Bar chart, worldwide: (**A**) VMF, (**B**) VMF excludes the possible anthropogenic dispersal data (VMF-e); (**C**) VMM; (**D**) VMN. (**E**,**F**) Pie chart, in China: (**E**) VMF; (**F**) VMM. area unit = 10^4^ km^2^. Abbr.: VM: *Vespa mandarinia*; VMF, *Vespa mandarinia* var. *magnifica*; VMM, *Vespa mandarinia* var. *mandarinia*; VMN, *Vespa mandarinia* var. *nobilis*.

Using the natural breaks method, the potential distribution of VMF was divided into four grades: viz. not suitable, marginally suitable, moderately suitable, and highly suitable areas. According to the optimized MaxEnt model, the distribution prediction maps of the suitable habitats for VMF, VMN, and VMM have been established, respectively (Figure 3 and Figure 4). The areas of highly suitable zones have been calculated and listed separately (Figure 5).

For VMF (Figure 3 and Figure 5A,E), the prediction indicates that the total area of highly suitable habitats reaches 260.59 × 10^4^ km^2^ (Figure 3). It mainly concentrates in Asia (247.38 × 10^4^ km^2^) especially in China (155.48 × 10^4^ km^2^). Next are Myanmar (30.00 × 10^4^ km^2^), India (17.89 × 10^4^ km^2^), Vietnam (12.88 × 10^4^ km^2^), Laos (12.23 × 10^4^ km^2^), Nepal (8.94 × 10^4^ km^2^), etc. Within China, VMF was mainly limited to the 800 mm isohyet, which, starting from the southeastern edge of the Qinghai–Tibet Plateau, turns northeast along the Min River basin, extends eastward across the main ridge line of the Qinling Mountains to reach the main stream of the Huai River, and finally terminates at the estuary of the Northern Jiangsu Irrigation Main Canal, serving as the boundary between the humid climate zone and semi-humid climate zone (Figure 2, notes 2 and 4). Yunnan has the largest area of highly suitable habitats (30.47 × 10^4^ km^2^), followed by Sichuan (22.29 × 10^4^ km^2^), Guangxi (16.43 × 10^4^ km^2^), Guizhou (15.57 × 10^4^ km^2^), Guangdong (12.73 × 10^4^ km^2^), Jiangxi (11.97 × 10^4^ km^2^), Hunan (10.64 × 10^4^ km^2^), etc. (Figure 3 and Figure 4B). Interestingly, the vast plains spanning in Henan, Hubei, Hunan, Anhui, and Zhejiang are not an optimal habitat for VMF (Figure 3). Additionally, coastal regions in China (Liaoning (0.017 × 10^4^ km^2^), Shandong (0.017 × 10^4^ km^2^), Jiangsu (0.39 × 10^4^ km^2^), Zhejiang (2.78 × 10^4^ km^2^), Taiwan (2.83 × 10^4^ km^2^), Hainan (0.94 × 10^4^ km^2^) etc.), India (17.89 × 10^4^ km^2^), North Korea (1.10 × 10^4^ km^2^), South Korea (3.20 × 10^4^ km^2^), and Japan (1.17 × 10^4^ km^2^) also have extensive suitable habitats (Figure 5E). Although VM has never penetrated into other large geographical regions (i.e., Europe, Australia, Africa, the Americas, etc.), the predictive model indicates that scattered suitable habitats are likely to exist in coastal regions such as Oceania (eastern coast of Australia (3.19 × 10^4^ km^2^) and New Caledonia (0.083 × 10^4^ km^2^), Southern Africa (South Africa (1.13 × 10^4^ km^2^), Malawi (0.26 × 10^4^ km^2^), Tanzania (0.122 × 10^4^ km^2^), and Mauritius (0.002 × 10^4^ km^2^) etc.), and the Americas (Mexico (1.60 × 10^4^ km^2^), Bolivia (0.34 × 10^4^ km^2^), Argentina (5.84 × 10^4^ km^2^), and Brazil (0.19 × 10^4^ km^2^) (Figure 5A).

To further investigate the potential suitable habitats of VMF under its early distribution pattern (*n* = 132), we excluded the possible expansion data (VMF-e, the new distribution record in China after 2014 when farmers began large-scale commercial export of VMF to the other provinces according to Huang et al. [20]), and then simulated and reconstructed its suitable habitats based on the current climatic data (Figure 5B). The results showed that the total area of highly suitable habitats of the whole world was only about 121.25 × 10^4^ km^2^. Among them, the area of Asia is 115.28 × 10^4^ km^2^, including the area of China: 45.026 × 10^4^ km^2^. It is evident that over the past 10 years, the predicted distribution range of VMF has more than doubled distinctly (121.25 × 10^4^ km^2^ vs. 260.29 × 10^4^ km^2^), especially in China (from 45.026 × 10^4^ km^2^ to 155.48 × 10^4^ km^2^; the area may increased by approximately 2.6×) (Figure 5B and Figure S2).

We further predicted the potential suitable habitats for VMN under current, which are considered transitional forms between VMF and VMM. The results show that a total area of highly suitable habitats is only about 5.95 × 10^4^ km^2^. Among them, the area of Asia is 4.18 × 10^4^ km^2^ (>70% of the world), including of China (2.98 × 10^4^ km^2^, >71% of Asia). Within China, besides the recorded distribution site in Taiwan, there might be highly suitable habitats in the coastal zones of Guangdong, Western Hainan. Outside China, Northwestern Vietnam may have quite large highly suitable habitats (Figure 4A and Figure 5D). Interestingly, outside Asia, North America and South America might have two very small highly suitable habitats, as well as Australia.

For VMM, the prediction shows the total area of highly suitable habitats reaches 225.57 × 10^4^ km^2^. It might be almost only in Asia (225.55 × 10^4^ km^2^), especially in China (178.31 × 10^4^ km^2^, more than VMF (155.48 × 10^4^ km^2^)). VMM extends further northwest along the 400 mm isohyet, forming continuous suitable regions in Hubei, Shaanxi, Shandong, Hebei, Liaoning etc. within China. Interestingly, to the west, there is a distinct curving boundary extending from the Western Sichuan Plateau to the eastern margin of the Hengduan Mountains, while to the south, the Nanling Mountains serve as the boundary. Outside China, most highly suitable habitats concentrated in North Korea, South Korea, Japan, and Russia in the Far East. Beyond that, it is almost impossible for dispersal to any other part of the world (Figure 4B and Figure 5C,F). Interestingly, a very small area (0.02 × 10^4^ km^2^) in USA is predicted to be the highly suitable habitats.

When comparing the prediction models of the three color forms, the results reveal that the distribution of VMF overlaps significantly with both VMM and VMN; however, there is almost no overlap between VMN and VMM. Obviously, VMF exhibits a broader ecological tolerance across multiple environmental dimensions than VMM and VMN. The potential dispersal geographic ranges of the three taxa are ranked as follows: VMF > VMN > VMM.

### 3.4. The Potential Distribution of VMF in Future Period

**Figure 6:**
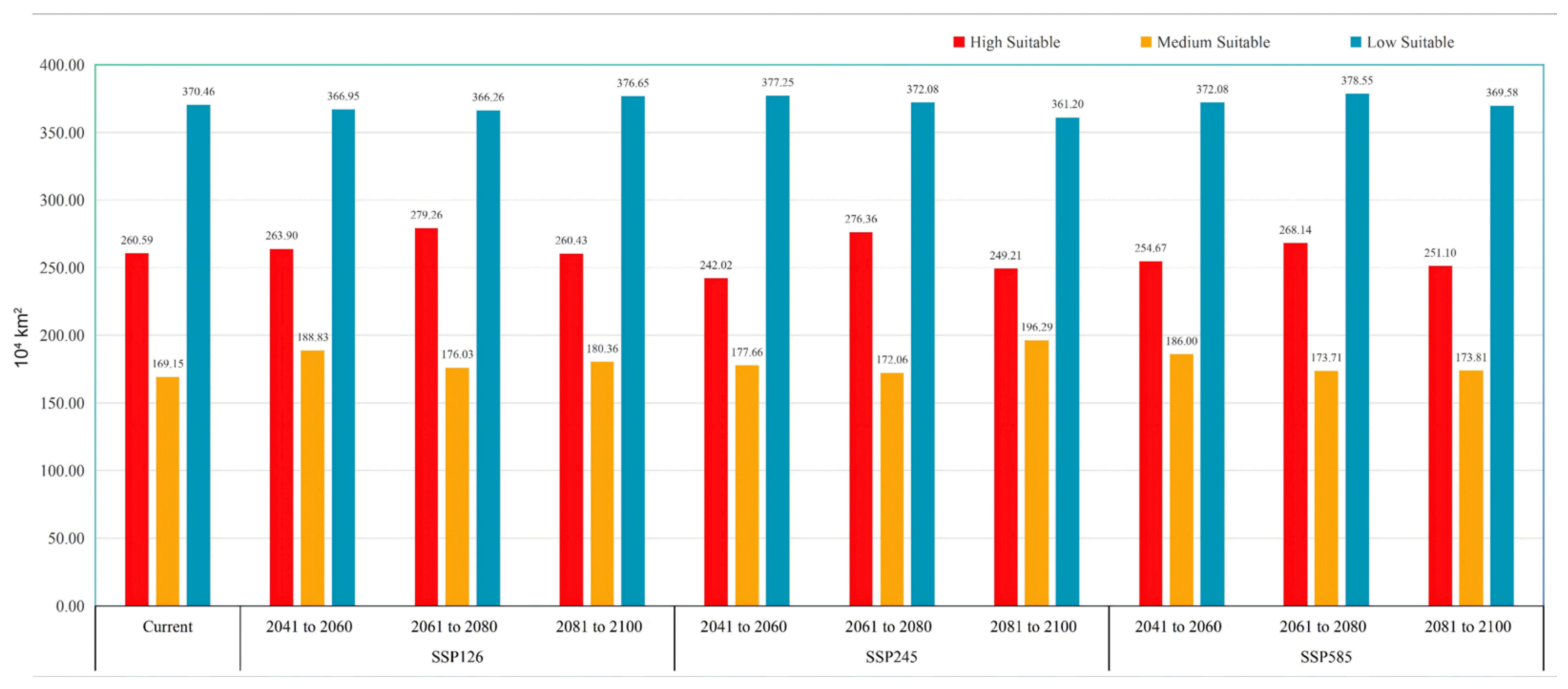
Predicted suitable areas for VMF under the current and future climatic conditions. Note: VMF, *Vespa mandarinia* var. *magnifica*. SSP126: “Green Road” scenario with low GHG emissions, sustainable development, and minimal climate mitigation/adaptation challenges; SSP245: “Middle of the Road” scenario with moderate GHG emissions, typical development, and medium climate mitigation/adaptation challenges; SSP585: Fossil-fueled scenario with high GHG emissions, energy-intensive growth, and major climate mitigation challenges.

Furthermore, we predicted the suitable habitats of VMF during 2041–2060, 2061–2080, and 2081–2100 under climate change scenarios SSP126 (low greenhouse gas emission scenario), SSP245 (medium greenhouse gas emission scenario), and SSP585 (high greenhouse gas emission scenario) (Figure 6 and Figure S3). Comparing to current projections, under SSP126, the numbers of highly suitable area (unit ×10^4^ km^2^) will continue increasing to 263.90 in 2041–2060 and 279.26 in 2061–2080, while they will decline to 260.43 in 2081–2100. Under SSP245, the changes are fluctuating, with a decrease to 242.02 in 2041–2060, a recovery to 276.36 in 2061–2080, and then a decline to 249.21 in 2081–2100. Under SSP585, the changes show minor variations, with a decline to 254.67 in 2041–2060, up to 268.14 in 2061–2080, and down to 251.10 in 2081–2100. The numbers of moderately suitable areas generally increase (from 172.06 to 196.29) under all scenarios, whereas the low suitable areas remain relatively stable (from 361.20 to 378.55).

Overall, under future climate change scenarios, SSP126 will be the most favorable for the expansion of VMF. During the period from 2081–2100, under all three scenarios, the area of highly suitable habitats will be smaller than the current size of 260.59 × 10^4^ km^2^ (Figure 6).

### 3.5. Relationship Between Climate Variables and Potential Distribution of VMF and VMM

**Figure 7:**
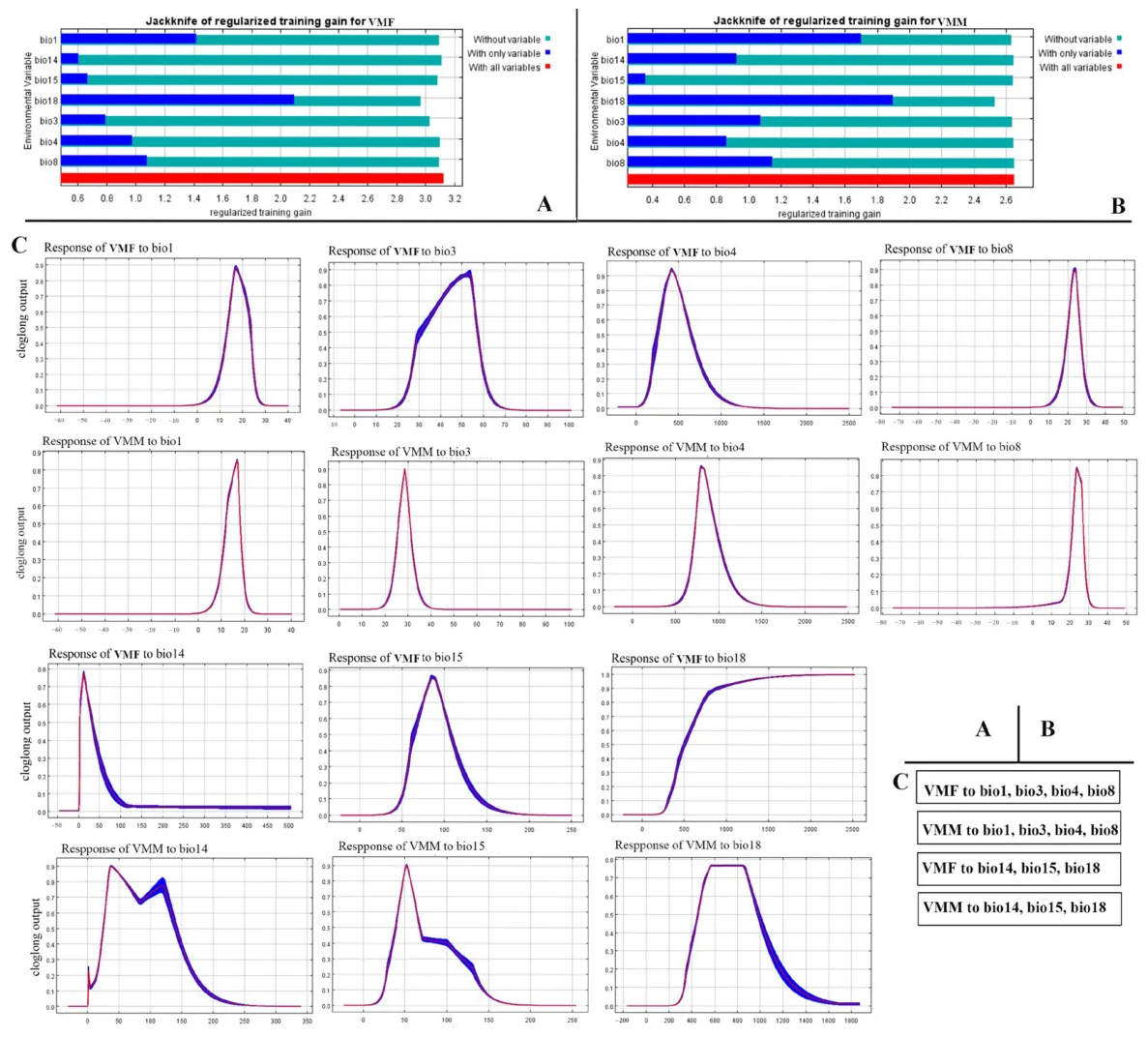
Estimating the value of major climate variables by Jackknife (**A**,**B**) and response curves of VMF and VMM to major climate variables (**C**). Abbr.: VMF, *Vespa mandarinia* var. *magnifica*; VMM, *V. mandarinia* var. *mandarinia*. Note. for (**A**,**B**): 1. Gain from training with only variable (blue bar): the gain obtained by running the model using this single variable alone, which directly reflects the independent predictive power of the variable itself. 2. Gain of remaining variables without this variable (green bar): the gain obtained by removing this variable from all environmental predictors and running the model with the rest of the variables. 3. Total gain from training with all variables (red bar): the baseline gain of the model when all variables are input, serving as a reference scale. for (**C**): The red curve and blue shading indicate the mean and the mean ± one standard deviation of the logistic probability of presence for the 100 MaxEnt replicate runs, respectively.

Using Maxent software (version 3.4.4) and the Jackknife method, we assessed the influence of the selected seven key climate factors (Bio1, Bio3, Bio4, Bio8, Bio14, Bio15, and Bio18) on the potential distribution of VMF and VMM (Figure 7). The results of Jackknife of the regularized training gain indicate that precipitation of warmest quarter (Bio18) and annual mean temperature (Bio1) have the most significant effect on model performance for VMF and VMM both (Figure 7A,B). Additionally, precipitation of driest month (Bio14) exerts are relatively greater impact on VMM than on VMF, as well as Isothermality (Bio3).

Figure 7C demonstrates that the response curves of climatic factors affect the highly suitable areas of VMF and VMM. For all temperature-related variables (Bio1, Bio3, Bio4, and Bio8), the curves of VMF and VMM exhibit a similar pattern: the occurrence probability increases continuously from zero to a peak, and then gradually declines back to zero as the climate index value increases. However, for precipitation-related variables (Bio14, Bio15, and Bio18), the curves of VMF and VMM exhibit different patterns, respectively. Response curve of VMF to Bio14 exhibits that the rising slope is distinctly steep; after reaching the peak (11, 0.78), the occurrence probability gradually declines to 0.03 and then remains stable. Meanwhile, the curve of VMM exhibits three peaks (2, 0.25) (37, 0.9), and (120, 0.78). Response curve of VMF to Bio15 is simple, similar to the upper temperature-related variables. However, the curve of VMM to Bio15 exhibits that the declining slope is maintaining a relatively stable level (0.41–0.4) at 70 mm–100 mm. For response curve of VMF to Bio18, the occurrence probability rises rapidly, then increases slowly to a peak of 0.98 at 1698 mm before plateauing. However, curve of VMM to Bio18 remains a stable peak of 0.76 at 572 mm–880 mm.

We compared the suitable range and optimum of VMF and VMM distribution influenced by major climatic factors with reference value of China in Table 1. The results indicate that the optimal distribution area for VMF is in a Tropical region, such as the Hainan, Xishuangbanna, and Subtropical regions in most of Southern China. It is worth noting that the suitable distribution range of all the major climactic factors for VMF are very broad, encompassing the north and northeast temperate regions, as well as reaching the edge of the Northwest Inner Dry land Region. Although, similar to VMF, most optimal values of VMM fall within the Tropical and Subtropical regions, their suitable range is relatively narrow. It can be concluded that the suitable habitat range of VMF is broader than VMM.

### 3.6. Analyzed the Multivariate Environmental Similarity Surface (MESS) and Most Dissimilar Variable (MOD) for VMF

**Figure 8:**
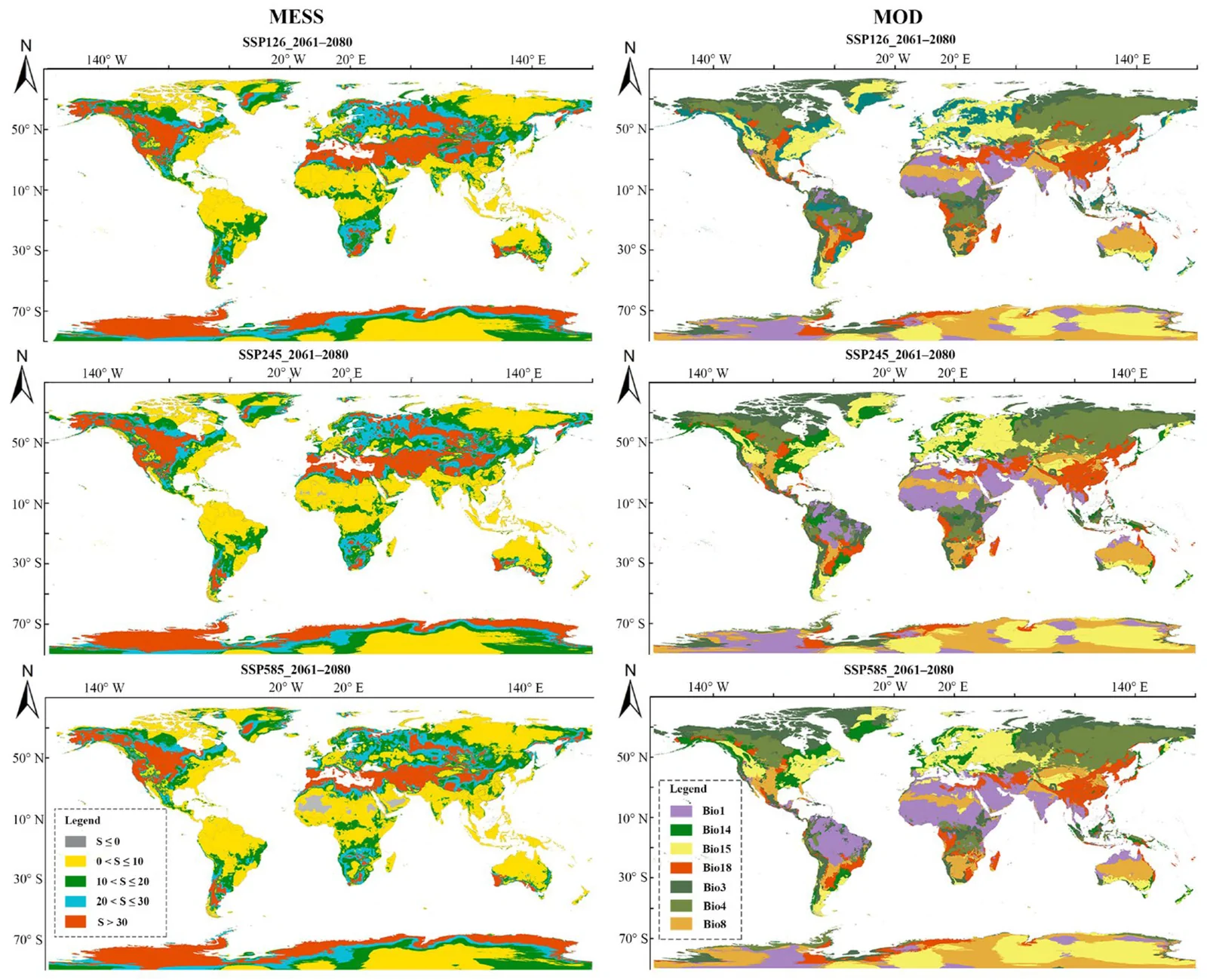
Analysis of MESS and MoD for VMF-suitable habitats under SSP126, SSP245, and SSP585 scenarios in 2061–2080. Abbr.: MESS, multivariate environmental similarity surface. MoD, Most dissimilar. s, similarity; VMF, *Vespa mandarinia* var. *magnifica*.

The study analyzed the multivariate environmental similarity and climate anomaly levels under different gas emission scenarios (SSP126, SSP245, and SSP585) to reveal the environmental change trends for VMF in world in the future. The results show that the mean values across the scenarios are quite close (around 2.45–2.15), indicating high multivariate environmental similarity and relatively small climate differences. The multiple similarity value was the lowest, and the degree of climate anomaly was the highest in the SSP585 across the 2070s climate scenario. In contrast, low-emission scenarios (SSP126 and SSP245) exhibit higher environmental similarity and more moderate impacts from climate change, with a lower risk of climate anomalies (Figure 8 and Figure S4).

Based on the MoD results, this study analyzed the primary driving factors of environmental changes in world under different emission scenarios (SSP126, SSP245, and SSP585). Precipitation seasonality (Bio15) was the dominant variable. It consistently had the highest contribution across all time periods. Under the SSP126 and SSP245 scenarios, the contribution of precipitation during the warmest quarter (Bio18) was also higher than that under other climate periods. Areas dominated by Bio18 spatially coincided with the current high suitability zones of VMF. Over time, the contribution of annual mean temperature (Bio1) increased steadily, reaching the highest proportion by the end of the century, whereas the influences of precipitation variables (Bio14 and Bio18) gradually decline. This opposing trend indicates a shift in primary drivers from precipitation dominated to temperature-dominated regimes under intensifying global warming. Clearly, this is also the reason why the size of highly suitable habitats will decline in 2081–2100. By contrast, isothermality (Bio3) exhibited the smallest variation across all scenarios and periods, suggesting its relatively low sensitivity to emission intensity (Figure 8 and Figure S5).

### 3.7. The Centroid Variation in the Potential Distribution of VMF

**Figure 9:**
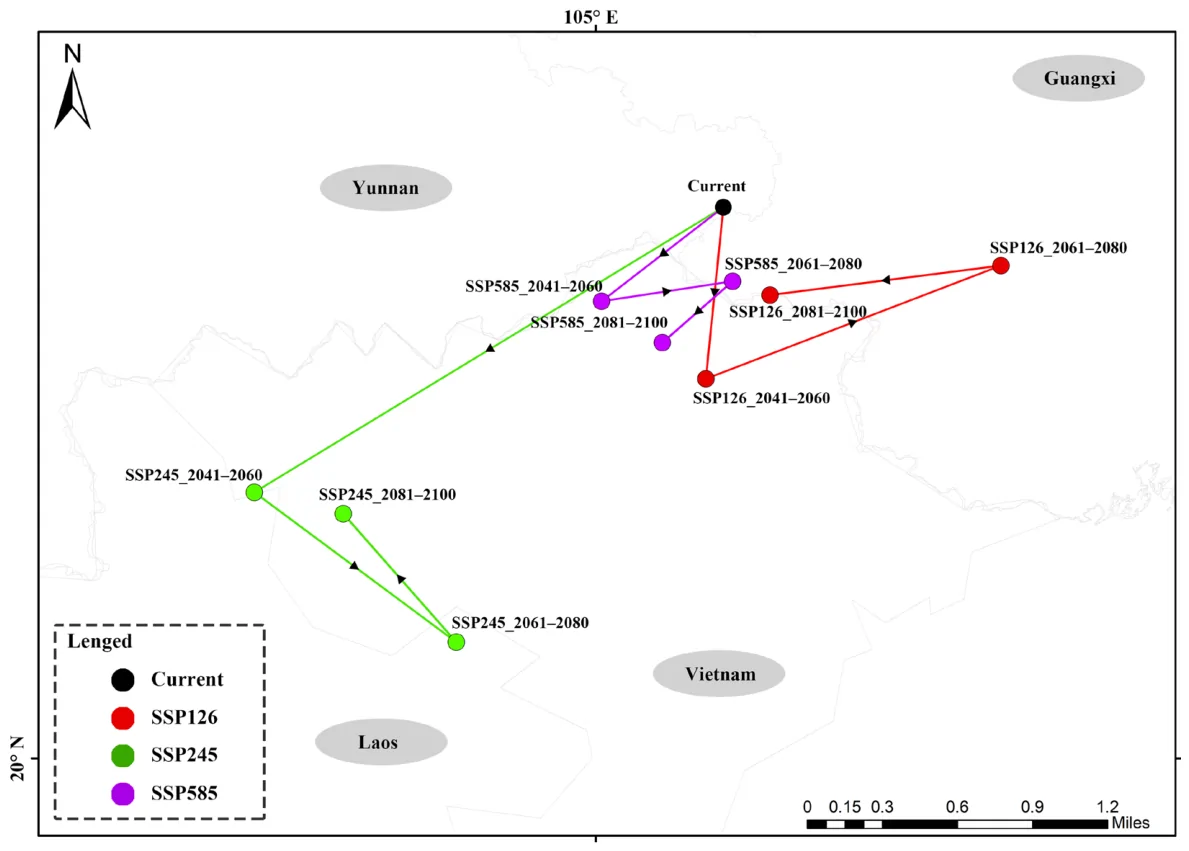
The change in the centroid of the potential distribution area of *V. mandarinia* var. *magnifica* (VMF) in China. Note: SSP126: “Green Road” scenario with low GHG emissions, sustainable development, and minimal climate mitigation/adaptation challenges; SSP245: “Middle of the Road” scenario with moderate GHG emissions, typical development, and medium climate mitigation/adaptation challenges; SSP585: Fossil-fueled scenario with high GHG emissions, energy-intensive growth, and major climate mitigation challenges. The black arrow (or triangle) points the migrating direction.

The migration trajectories of the suitability centroid for VMF under different climate scenarios are presented below. Currently, the centroid of the high-suitability area is located in Wenshan City, Yunnan Province. Under the SSP126 scenario, the centroid shifted southwestward in the early century (with the centroid located in Vietnam), but from the 2080s to the 2100s, the trajectory was dominated by a pronounced southeastward shift (with the centroid located in Nanning or Baise City, Guangxi Zhuang Autonomous Region). Under the SSP245 scenario, the centroid is projected to shift 229.38 km southwestward into Vietnam, with the strongest southwestward movement occurring by the 2060s. Under the SSP585 scenario, the centroid shifted southwestward in the 2060s and 2100s (with the centroid located in Vietnam), but the trajectory was dominated by a southeastward shift in the 2080s (with the centroid located in Baise City, Guangxi Zhuang Autonomous Region). In summary, these results indicate that although migration trajectories differ in complexity across different scenarios, the long-term distribution trend of this species under climate change is consistently southeastward, with the most marked shifts occurring under low-emission conditions (Figure 9).

## 4. Discussions

By employing geological-climatic models and biodiversity models to conduct analytical research on more than 20,000 vertebrate species, Skeels et al. [36] uncovered the underlying mechanism of the species from the Oriental realm, which successfully colonized Australia after crossing the Wallace Line, while species from the Australian realm failed to cross this biogeographic boundary. This study concludes that broad precipitation tolerance and dispersal ability are the core determinants facilitating species exchange across the deep-time precipitation gradient in this region [36]. Interestingly, our species distribution prediction model indicates that the distribution patterns of the giant hornet polymorphism *Vespa mandarinia* var. *magnifica* (VMF) and *Vespa mandarinia* var. *mandarinia* (VMM) in China also appear to support this conclusion.

China spans the Oriental and Palaearctic zoogeographic realms. However, no consensus has been reached on understanding the western boundary between the two realms [37,38,39]. The determination of zoogeographical divisions, especially on the specific boundary, depends on the distribution patterns of the animals to be discussed. Hoffmann (2001) [38] proposes a boundary of the Palaearctic–Oriental transitional zone (between 33° N and 28° N) as defined for mammals. This result is challenged by many Chinese researchers for mammals with high adaptation to environment diversity and higher loco-motion ability [37]. In our research, a para-“Wallace Line” is tentatively proposed to exist within China—specifically, the convergence boundary formed after the collision between the Indian Plate and the Eurasian Plate, roughly located at the east edges of the Western Sichuan Plateau and the Hengduan Mountains. VMM, which is adapted to the relatively dry climate of Northern China, rarely crosses back this line to disperse into the humid southern regions, whereas VMF, which originated in Southwestern China, has evolved deep adaptations to arid climates and has become widely dispersed across the region. The Nanling Mountains stretching across Southern China may partially obstruct the southward dispersal of VMM. However, numerous low-altitude (200–500 m) passes in between can also serve as dispersal corridors for the giant hornet *Vespa mandarinia* VM. In a certain sense, VMF and VMM might serve as a candidate model for studying the intrinsic mechanisms underlying the asymmetry of global biogeographic patterns and offer a tentative perspective on the west Palaearctic–Oriental boundary. Additionally, We should mention here, our hypothesis is based on spatial distribution data. The result may also reflect sampling gaps, historical collection effort, or environmental gradients unrelated to a true biogeographic barrier. Further validation with phylogenetic or gene-flow evidence is required to confirm whether this barrier represents a historically significant isolating mechanism.

Darlington mentioned that the main pattern of the dispersal of Vertebrates shows evolution of successive dominant groups in the great favourable area of the main part of the Old-World tropics and spread into smaller and for less favourable areas [40]. van der Vecht concluded that Vespinae have evolved in Southeastern Asia and radiated from there, but they did not penetrate into Australia or the tropics of Africa and America [41]. Using climatic suitability models, Villemant et al. [42]. assessed the potential invasion risk of the hornet alien bee-hawking Yellow-legged hornet *Vespa velutina* var. *nigrithorax* and well predicted its potential invasion range across all continents [42]. Compared with *Vespa velutina*, VM is mainly native in Asia. Accidentally, the largest stinging social insect species, VM, was introduced into North America in 2019 and reported from Marysville, Washington, on 4 June 2021. People worried that it could threaten honeybees and sting humans. However, our prediction results indicated that most regions of USA are not suitable habitats for VM. The distribution prediction results can also be used to assess the intrusion risk of VM in other regions.

Local adaptation of animal colouration is crucial in the processes of adaptive evolution. Genetically differentiated color morphs in nature exhibit specific distribution areas, and this phenomenon is affected by a combination of multiple factors. Our research proposed four main color forms of VM, including a new one, VMP. Overall, the color patterns of VM exhibit a gradient trend from southwest to northeast (from VMF to VMM), where the terminal band widens as latitude increases. At the intersecting boundary of the two color pattern distributions, some transitional individuals, viz. VMM-t, are observed, and these individuals bear striking resemblance to the characteristics of VMN distribution in Taiwan. Given that hybrid breeding may generate transitional morphological forms, we deliberately refrained from definitively classifying VMM-t as an independent color morph. Obtaining additional specimens accompanied by nesting information and molecular data is expected to resolve this question. Geographically, Taiwan is located southeast of this convergence zone. Based on this observation, we infer that the VMN in Taiwan may originate from this transitional zone. The geographical isolation by the Taiwan Strait may have hindered genetic exchange between populations. Using the molecular data online, our research group attempted to investigate the maximum distance across the strait that VM can traverse. Preliminary results indicate that a distance of 60 km cannot prevent stable gene flow, whereas the likelihood of genetic exchange drops significantly at distances beyond 100 km (unpublished data). Further collection and molecular research will be essential to truly solve the problem on distribution of VM. This gradual morphological transition offers a novel morphogeographic perspective for clarifying the long-standing taxonomic ambiguity associated with the varities of *Vespa mandarinia*.

It is well known that contrasting colors, such as the yellow and black stripes of social wasps, are typical aposematic signals. Surprisingly, hornets commonly have a geographic color variation through melanic polymorphism, potentially driven by different selective pressures. Some researchers analyzed 11 geographic variations (among them, only four in China) of *Vespa velutina* (Hymenoptera: Vespidae) [43]. The results showed that melanism in populations is more likely influenced by constraints on aposematism and Mullerian mimicry than by abiotic pressures.

In general, the color forms of VM agree with Gloger’s rule (darker colouration occurs more often in wet and warm environments) [44,45]. Subtropical and moisture areas such as Southeast Asia are the ideal place for VMF, while the subtropical but dry areas, such as Pakistan and Western India, are fitting for the VMP color form. A temperate, dry continental climate with relatively low temperatures is fitting for VMM. Irregular small ferruginous patches on mesonotum of several individuals are possibly constrained by the microclimate. Obviously, VM has a strategy completely different from *V. velutina*; its populations are more likely influenced by climatic constraints.

Hornets in the genus *Vespa* are large, predatory, eusocial wasps native to Europe and Asia. As predators of already declining populations of native insects, spiders, and especially honeybees, some of the introduced *Vespa* species have shown to be environmental stressors and could be competitors for food and nesting sites of native wasps and impact human safety because of their aggressiveness and fatal reactions to their venom [19,46,47].

As the country with the highest species diversity of the genus *Vespa*, China faces a severe problem of hornet attacks [48]. Multiple hornet stinging is a frequent medical emergency in the mountainous areas, characterized by rapid onset and high mortality rates. Especially from July–September 2013 alone, 1685 cases of stung persons and 42 deaths were reported in the Qinba mountain area in Shaanxi, which caused a significant public outcry both domestically and internationally [48,49,50,51].

However, the hornets, especially the giant VMF (commonly named “Hongniang”), have been successfully reared for their delicious-tasting larvae and potential medicinal value, and they have rapidly spread in China since 2014. Huang et al. reported that 657 breeding groups have been registered, of which more than half (57%) are in Yunnan, and 95% are in Southern China [20].

Compared with its congener, VMF is much larger and poses a greater threat to bees and humans. Hornet farmers have mastered breeding techniques for the supersized VMF, with multiple queens building one nest. The unregulated introduction of this significantly larger hornet may threaten local biodiversity. Our research has clearly identified suitable habitats for both VMM and VMF.

Since VMF-e contains novel distribution records collected after 2014, it is difficult to determine whether the population has reached distributional equilibrium after a decade. To address this issue, we constructed two modeling scenarios: the maximum scenario, including the VMF-e dataset, and the minimum scenario excluding it. Comparing the results of these two scenarios can clearly demonstrate the magnitude of the species’ range expansion. In the following analysis, we focus on the results from the maximum scenario with VMF-e included, while the actual distribution status is likely to fall between the results of the two scenarios.

Notably, within the past decade, anthropogenic activities and unregulated field releases in China have facilitated a significant expansion of VMF distribution beyond its native range, with its suitable distribution area increasing rapidly to its current maximum. This expansion coincides with the observed northward shift of precipitation belts in recent decades. Under future climate scenarios (SSP126, SSP245, and SSP585), our projections show that the area of highly suitable habitat for VMF will experience slow fluctuations and will decline relative to the current extent by the period of 2081–2100.

Although we repeatedly warn about the unregulated release of the “Hongniang” phenotype, we have to admit that no formal risk assessment, like pathway analysis, propagule pressure estimation, or economic impact assessment, could be provided in this study. Even though the distribution models show suitable habitat expansion, this does not directly equate to invasion risk. The following research on quantifying the factors such as human-mediated transport, establishment probability, and impact on native species are expected.

This is the first study globally using environmental factors to estimate the potential distribution and dispersal of *Vespa mandarinia*. Despite the strong predictive performance and broad applicability of the MaxEnt model in forecasting potential species distributions, certain limitations in its application to invasion risk prediction (e.g., niche conservatism, sampling bias, and equilibrium assumption) still exist [52]. For example, when future species distributions are predicted by a model using only a single General Circulation Model (GCM, (BCC-CSM2-MR), the estimated potential species range may be incomplete or misleading [53,54,55]. Although the present study follows the conventional validation protocol for MaxEnt modeling, modeling across large geographic regions still carries the risk of sampling bias [56]. The global background sampling approach adopted in this study leverages the full spectrum of global climatic variation as a reference, but it may introduce additional uncertainties into species distribution predictions at the regional scale. Ecological indicators merely characterize the fundamental ecological requirements of a species, rather than reflecting its realized ecological niche. Additionally, when applying species distribution models to predict potential species distributions, multiple biotic and abiotic drivers that shape species distribution patterns are frequently omitted from the analysis [57,58,59]. Consistently, this study also did not incorporate edaphic properties, crop distribution, predators, anthropogenic activities, and other relevant environmental factors into the modeling framework [60,61]. Future improvements can be achieved through adopting multi-model ensembles, setting region-specific background points, conducting follow-up field surveys, establishing long-term continuous monitoring of population dynamics, and integrating economic and social indicators into predictive frameworks.

## 5. Conclusions

This study presents the first global systematic analysis of the color polymorphism and distribution pattern of the giant hornet *Vespa mandarinia* (VM). A total of 42 images covering all documented color phenotypes were examined, and the identified morphs were classified into four primary color varieties, including one newly described variety: *Vespa mandarina* var. *pseudomagnifica* (VMP). More importantly, species distribution modeling indicates that VM is primarily native to Asia, with the largest suitable habitat concentrated in China. The “400 mm isohyet” in China acts as a major biogeographic barrier limiting the northwestward range expansion of VM. The Western Sichuan Plateau and Hengduan Mountains were tentatively proposed as a para-“Wallace Line” within China, which shapes the biogeographic distribution of *Vespa mandarinia* var. *magnifica* (VMF) and *Vespa mandarinia* var. *mandarinia* (VMM), forming a distinct asymmetrical biogeographic pattern and highlight the west Palaearctic–Oriental boundary.

## Figures and Tables

**Table 1:** Comparison of the optimal ranges for climate variables under the potential distributions of VMF and VMM.

Climate Variables	Suitable Range		Optimum Value	
	VMF	VMM	VMF	VMM
Bio1 (°C)	−5–30	−3–27	24	24
Bio3 (°C)	10–79	13–46	54	29
Bio4 (°C × 100)	14–1664	254–1789	421	787
Bio8 (°C)	−0.9–39	−31–35	24	24
Bio14 (mm)	0.4–459	1–307	11	2; 37; 120
Bio15 (mm)	19–220	3–208	85	51
Bio18 (mm)	137–1691	209–1869	1691	572–880

Note: The data in the table have been rounded. Numbers greater than 1 do not retain decimal places. Abbr. VMF, *Vespa mandarinia* var. *magnifica*; VMM, *V. mandarinia* var. *mandarinia*. Bio1 Annual mean temperature (°C); Bio3 Isothermality (Bio2/Bio7) × 100; Bio4 Temperature seasonality (standard deviation × 100); Bio8 Mean temperature of wettest quarter (°C); Bio14 Precipitation of driest month (mm); Bio15 Precipitation seasonality (mm) (Coefficient of Variation); Bio18 Precipitation of warmest quarter (mm).

## Data Availability

All data generated from this study were included in the current manuscript.

## Supplementary Materials

The following supporting information can be downloaded at: https://www.mdpi.com/article/10.3390/insects17090934/s1, Figure S1: ROC curve of potential distribution modelling results for VMF; Figure S2: The suitable habitat distribution of the early-stage VMF in China exhibits notable regional variations under current climatic conditions; Figure S3: A predictive map of potentially suitable areas for VMF in world under different climate change scenarios; Figure S4: Multivariate environmental similarity surface (MESS) for VMF under SSP126, SSP245, and SSP585 in 2041–2060 and 2081–2100; Figure S5: Most dissimilar (MoD) for VMF SSP126, SSP245, and SSP585 in 2041–2060 and 2081–2100; Table S1: Environmental variables and their descriptions used for MaxEnt modeling in VMF; Table S2: The Pearson correlation coefficient between each environmental factor for VMF; Table S3: Predicted suitable areas for VMF under the current and future climatic conditions.

## References

[1] Endler J.A. (1980). Natural selection on color patterns in Poecilia reticulata. Evolution.

[2] Lu Y.Y., Kovalev A., Li L.L., Li C.C., Zhu X.Y., He M., Yang X.K., Bai M., Gorb S.N. (2025). Structural background of intraspecific color polymorphism and the driver of geographic patterns in a shining leaf chafer. Front. Zool..

[3] Mills L.S., Bragina E.V., Kumar A.V., Zimova M., Lafferty D.J.R., Feltner J., Davis B.M., Hackländer K., Alves P.C., Good J.M. (2018). Winter color polymorphisms identify global hot spots for evolutionary rescue from climate change. Science.

[4] Sun B.J., Li W.M., Lv P., Wen G.N., Wu D.Y., Tao S.A., Liao M.L., Yu C.Q., Jiang Z.W., Wang Y. (2024). Genetically Encoded Lizard Color Divergence for Camouflage and Thermoregulation. Mol. Biol. Evol..

[5] Gray S.M., McKinnon J.S. (2007). Linking color polymorphism maintenance and speciation. Trends Ecol. Evol..

[6] Takahashi Y., Noriyuki S. (2019). Colour polymorphism influences species’ range and extinction risk. Biol. Lett..

[7] Laumeier R., Brändle M., Rödel M.O., Brunzel S., Brandl R., Pinkert S. (2023). The global importance and interplay of colour-based protective and thermoregulatory functions in frogs. Nat. Commun..

[8] Jia Z.A., Fernandes M.C., Deng Z.F., Yang T., Zhang Q.T., Lethbridge A., Yin J., Lee J.H., Han L., Weaver J.C. (2021). Microstructural design for mechanical–optical multifunctionality in the exoskeleton of the flower beetle Torynorrhina flammea. Proc. Natl. Acad. Sci. USA.

[9] Smith F. (1852). Descriptions of some new and apparently undescribed species of Hymenopterous insects from North China, collected by Robert Fortune, Esq. Trans. Entomol. Soc. Lond..

[10] Sonan J. (1929). On Vespa from Formosa. Trans. Nat. Hist. Soc. Formosa.

[11] Smith F. (1852). Descriptions of some Hymenoterous insects captured in India, with notes on their economy, by Ezra T. Downes, Esq. who presented them to the Honourable the East India Company. Ann. Mag. Nat. Hist..

[12] Smith F. (1871). Descriptions of some new insects collected by Dr. Anderson during the Expedition to Yunnan. Proc. Zool. Soc. Lond..

[13] Cameron P. (1903). Descriptions of four species of Vespa from Japan. Entomologist.

[14] Radoszkowski O. (1857). Entomolgie speciale Insectes du Japon. Etudes Entomol. Hels..

[15] du Buysson R. (1905). Monographie des guêpes ou *Vespa*. Ann. Soc. Entomol. Fr..

[16] Matsumura S. (1930). The Illustrated Thousand Insects of Japan, II (Hymenoptera).

[17] Archer M.E. (1995). Taxonomy, distribution and nesting biology of the *Vespa mandarinia* group (Hym., Vespinae). Entomol. Mon. Mag..

[18] Carpenter J.M., Kojima J. (1997). Checklist of the species in the subfamily Vespinae (Insecta:Hymenoptera:Vespidae). Nat. Hist. Bull. Ibaraki Univ..

[19] Smith-Pardo A.H., Carpenter J.M., Kimsey L. (2020). The Diversity of Hornets in the Genus *Vespa* (Hymenoptera: Vespidae; Vespinae), Their Importance and Interceptions in the United States. Insect Syst. Divers..

[20] Huang X.Q., Yang Y.X., Hu Z.W., Miao C.H., Liang C., Lu H.X. (2018). Analysis of the development of Hornets breeding in Yunnan Province. Apical True China.

[21] Kim D.E., Lee H., Kim M.J., Lee D.H. (2015). Predicting the potential habitat, host plants, and geographical distribution of *Pochazia shantungensis* (Hemiptera: Ricaniidae) in Korea. Korean J. Appl. Entomol..

[22] Elith J., Leathwick J.R. (2009). Species distribution models: Ecological explanation and prediction across space and time. Ann. Rev. Ecol. Evol. Syst..

[23] Poutsma J., Loomans A.J.M., Aukema B., Heijerman T. (2008). Predicting the potential geographical distribution of the harlequin ladybird, *Harmonia axyridis*, using the CLIMEX model. BioControl.

[24] Lu S.S., Takahashi J., Yeh W.C., Lu M.L., Huang J.Y., Lin Y.J., Sung I.H. (2021). Evidence for range expansion and origins of an invasive hornet Vespa bicolor (Hymenoptera, Vespidae) in Taiwan, with notes on its natural status. Insects.

[25] Bessa A.S., Carvalho J., Gomes A., Santarém F. (2016). Climate and land-use drivers of invasion: Predicting the expansion of Vespa velutina nigrithorax into the Iberian Peninsula. Insect Conserv. Divers..

[26] Archer M.E. (2012). Vespine Wasps of the World.

[27] Van der Vecht J. (1959). Notes on Oriental Vespinae, including some species from China and Japan (Hymenoptera, Vespidae). Zool. Meded..

[28] Sharma R., Khan S., Kaul V. (2023). Predicting the potential habitat suitability and distribution of “Weed-Onion” (*Asphodelus tenuifolius* Cavan.) in India under predicted climate change scenarios. J. Agric. Food Res..

[29] Zhuo Z., Xu D., Pu B., Wang R., Ye M. (2020). Predicting distribution of *Zanthoxylum bungeanum* Maxim. in China. BMC Ecol..

[30] Wang R., Li Q., Feng C., Shi Z. (2017). Predicting potential ecological distribution of *Locusta migratoria tibetensis* in China using MaxEnt ecological niche modeling. Acta Ecol. Sin..

[31] Walden-Schreiner C., Leung Y., Kuhn T., Newburger T., Tsai W. (2017). Environmental and managerial factors associated with pack stock distribution in high elevation meadows: Case study from Yosemite National Park. J. Environ. Manag..

[32] Iturbide M., Gutierrez J.M., Alves L.M., Bedia J., Cerezo-Mota R., Cimadevilla E. (2020). An update of IPCC climate reference regions for subcontinental analysis of climate model data: Definition and aggregated datasets. Earth Syst. Sci. Data.

[33] Liu C.R., Newell G., White M. (2016). On the selection of thresholds for predicting species occurrence with presence-only data. Ecol. Evol..

[34] Elith J., Kearney M., Phillips S. (2010). The art of modelling range-shifting species. Methods Ecol. Evol..

[35] Wang X., Li Z., Zhang L., Wang Y., Liu Y., Ma Y. (2024). The optimized Maxent model reveals the pattern of distribution and changes in the suitable cultivation areas for *Reaumuria songarica* being driven by climate change. Ecol. Evol..

[36] Skeels A., Boschman L.M., McFadden I.R., Joyce E.M., Hagen O., Jiménez Robles O., Bach W., Boussange V., Keggin T., Jetz W. (2023). Paleoenvironments shaped the exchange of terrestrial vertebrates across Wallace’s Line. Science.

[37] Chen L., Song Y.L., Xu S.F. (2008). The boundary of palaearctic and oriental realms in western China. Prog. Nat. Sci..

[38] Hoffmann R.S. (2001). The southern boundary of the palaearctic realm in China and adjacent countries. Acta Zool. Sin..

[39] Tan J.L., Carpenter J.M., van Achterberg C. (2018). Most northern Oriental distribution of *Zethus Fabricius* (Hymenoptera, Vespidae, Eumeninae), with a new species from China. J. Hymenopt. Res..

[40] Darlington P.J. (1966). Zoogeography: The Geographical Distribution of Animals.

[41] Van der Vecht J. (1965). The geographical distribution of the social wasps (Hymenoptera: Vespidae). Pacific Island and Tropical themes. Proc. XII Int. Congr. Entomol..

[42] Villemant C., Barbet-Massin M., Perrard A., Muller F., Gargominy O., Jiguet F. (2011). Predicting the invasion risk by the alien bee-hawking Yellow-legged hornet *Vespa velutina nigrithorax* across Europe and other continents with niche models. Biol. Conserv..

[43] Perrard A., Arca M., Rome Q., Muller F., Tan J.L., Bista S. (2014). Geographic variation of melanisation patterns in a hornet species: Genetic differences, climatic pressures or aposematic constraints?. PLoS ONE.

[44] Delhey K. (2019). A review of Gloger’s rule, an ecogeographical rule of colour: Definitions, interpretations and evidence. Biol. Rev..

[45] Lev-Yadun S. (2015). Gloger’s rule in plants: The species and ecosystem levels. Plant Signal. Behav..

[46] Abrol D.P. (1994). Ecology, behaviour and management of social wasp, *Vespa velutina* Smith (Hymenoptera: Vespidae), attacking honeybee colonies. Korean J. Apicul..

[47] Choi M.B., Martin S.J., Lee J.W. (2011). Distribution, spread, and impact of the invasive hornet *Vespa velutina* in South Korea. J. Asia-Pac. Entomol..

[48] Tan J.L., van Achterberg C., Chen X.X. (2015). Potentially Lethal Social Wasps, Fauna of the Chinese Vespinae (Hymenoptera: Vespidae).

[49] Chen P., Yao W., Gong R., Ling R.J., Sun Y.H., Sun Y.W. (2022). Early intervention (≤6h) reduces the degree of multiple organ dysfunction with wasps sting patients. Minerva Med..

[50] Ling R.J., Yang X.Y., Xiao M. (2018). Expert consensus statement on standardized diagnosis and treatment of wasp sting in China. Chin. Crit. Care Med..

[51] Tan J.L., Xing L.X. (2021). Dengxian Shide Hufeng Mian, Illustration of Common Social Wasps and the Management on Their Attacking.

[52] Maruthadurai R., Das B., Ramesh R. (2023). Predicting the invasion risk of rugose spiraling whitefly, *Aleurodicus rugioperculatus*, in India based on CMIP6 projections by MaxEnt. Pest Manag. Sci..

[53] Gholami H., Lotfirad M., Ashrafi S.M., Biazar S.M., Singh V.P. (2023). Multi-GCM ensemble model for reduction of uncertainty in runoff projections. Stoch. Environ. Res. Risk Assess..

[54] Lee S., Qi J.Y., McCarty G.W., Yeo I.Y., Zhang X.S., Moglen G.E., Du L. (2021). Uncertainty assessment of multi-parameter, multi-GCM, and multi-RCP simulations for streamflow and non-floodplain wetland (NFW) water storage. J. Hydrol..

[55] Van de Vuurst P., Gohlke J.M., Escobar L.E. (2025). Future climate change and the distributional shift of the common vampire bat, *Desmodus rotundus*. Sci. Rep..

[56] Fourcade Y., Engler J.O., Rödder D., Secondi J. (2014). Mapping species distributions with MAXENT using a geographically biased sample of presence data: A performance assessment of methods for correcting sampling bias. PLoS ONE.

[57] Aidoo O.F., Amaro G.C., Souza P.G.C., Picanço M.C., Awuah-Mensah K.A., Silva R.S.D. (2025). Climate change impacts on worldwide ecological niche and invasive potential of Sternochetus mangiferae. Pest Manag. Sci..

[58] Ma L., Liu X., Chai J., Wang Y., Yang J. (2021). Effects of slope aspect and rainfall on below ground deep fine root traits and above ground tree height. Front. Plant Sci..

[59] Pereira P.S., Peterson J.A., Ramos R.S., Swoboda Bhattarai K.A., Picanço M.C., Sarmento R.A. (2026). Modeling of suitable geographic areas for Striacosta albicosta in corn and dry bean crops under climate change scenarios. Pest Manag. Sci..

[60] Wisniewski P., Märker M. (2021). Comparison of topsoil organic carbon stocks on slopes under soil-protecting forests in relation to the adjacent agricultural slopes. Forests.

[61] Zhao H., Xian X., Guo J., Yang N., Zhang Y., Chen B.X., Huang H.K., Liu W.X. (2023). Monitoring the little fire ant, Wasmannia auropunctata (Roger 1863), in the early stage of its invasion in China: Predicting its geographical distribution pattern under climate change. J. Integr. Agric..

