# Supplementary material for: Global Color Variation in Giant Hornet Vespa mandarinia Smith (Hymenoptera: Vespidae) and Potential Threat of Spread of the Commercially Reared “Hongniang” Phenotype
Source: Insects. 2026 Sep 7;17(9):934. doi: 10.3390/insects17090934 (PMC13607508; doi:10.3390/insects17090934)
Supplement: Supplementary file 1 [file insects-17-00934-s001.zip › insects-4528830-supplementary.pdf]

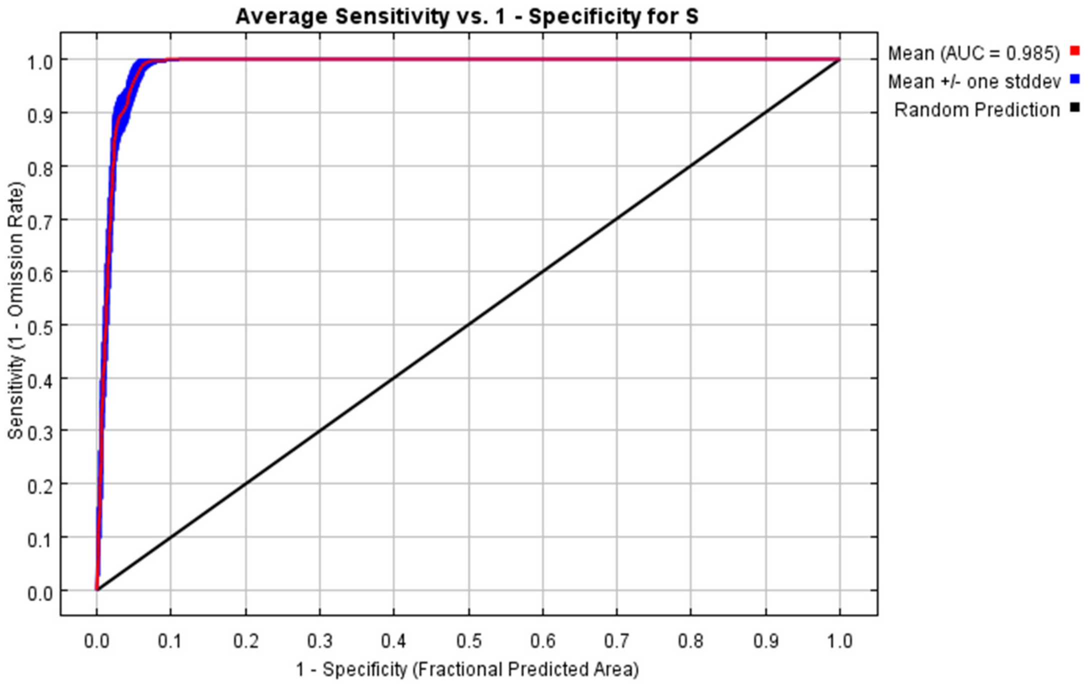

Figure S1. ROC curve of potential distribution modelling results for VMF.

Note: Red curve: mean sensitivity; blue shading: ± one SD. Black dashed line: random prediction (AUC = 0.5). Mean test AUC = 0.985, indicating excellent predictive performance.

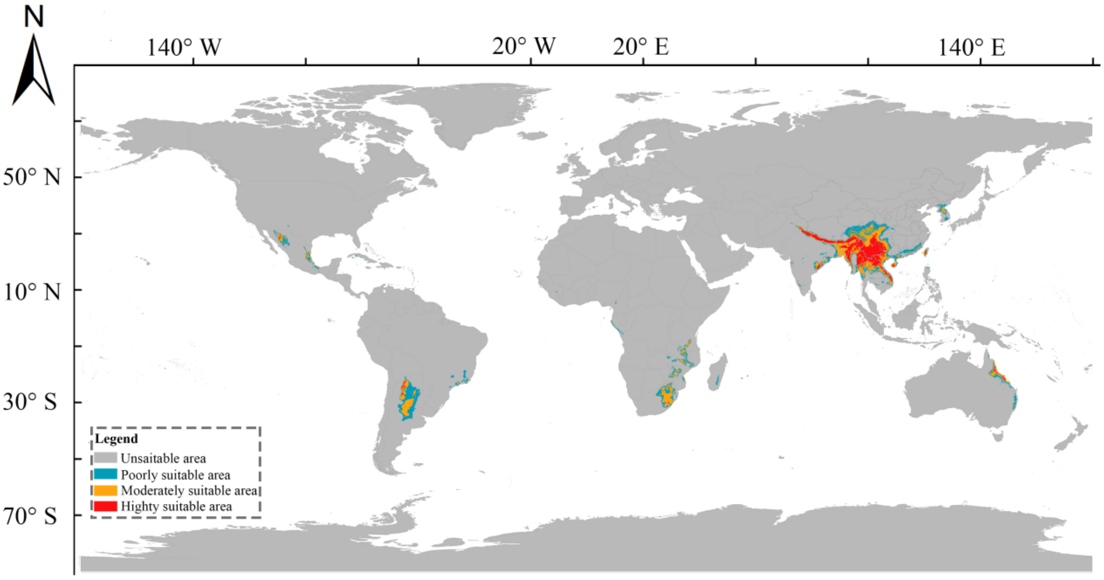

Figure S2. The suitable habitat distribution of the early-stage VMF in China exhibits notable regional variations under current climatic conditions. (Red: high suitability zone; orange: moderate suitability zone; blue:low suitability zone; grey: unsuitable zone).

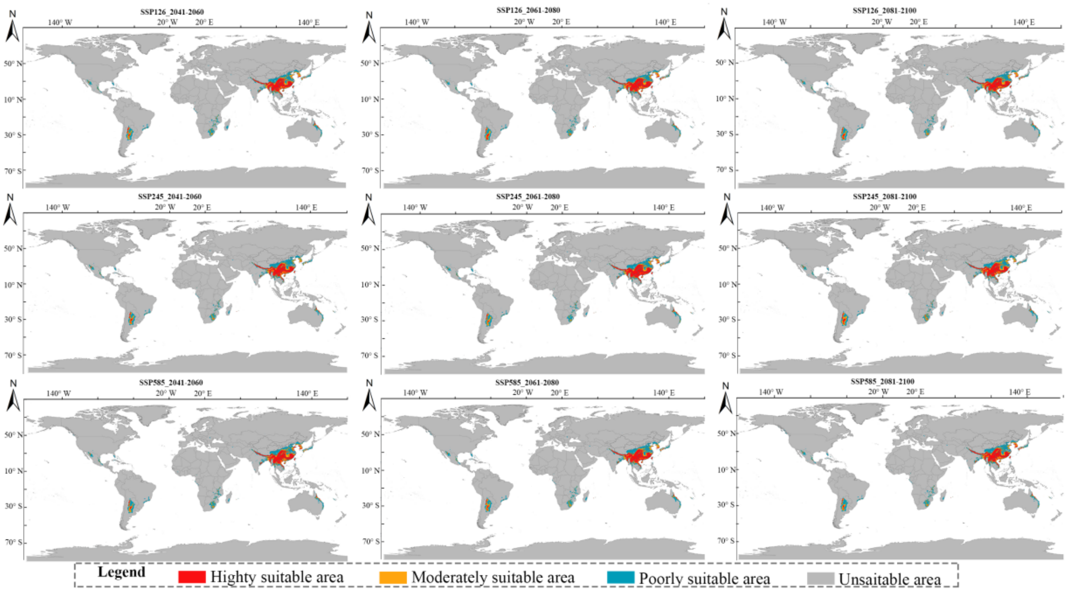

Figure S3. A predictive map of potentially suitable areas for VMF in world under different climate change scenarios. (Red: high suitability zone; orange: moderate suitability zone; blue:low suitability zone; grey: unsuitable zone).

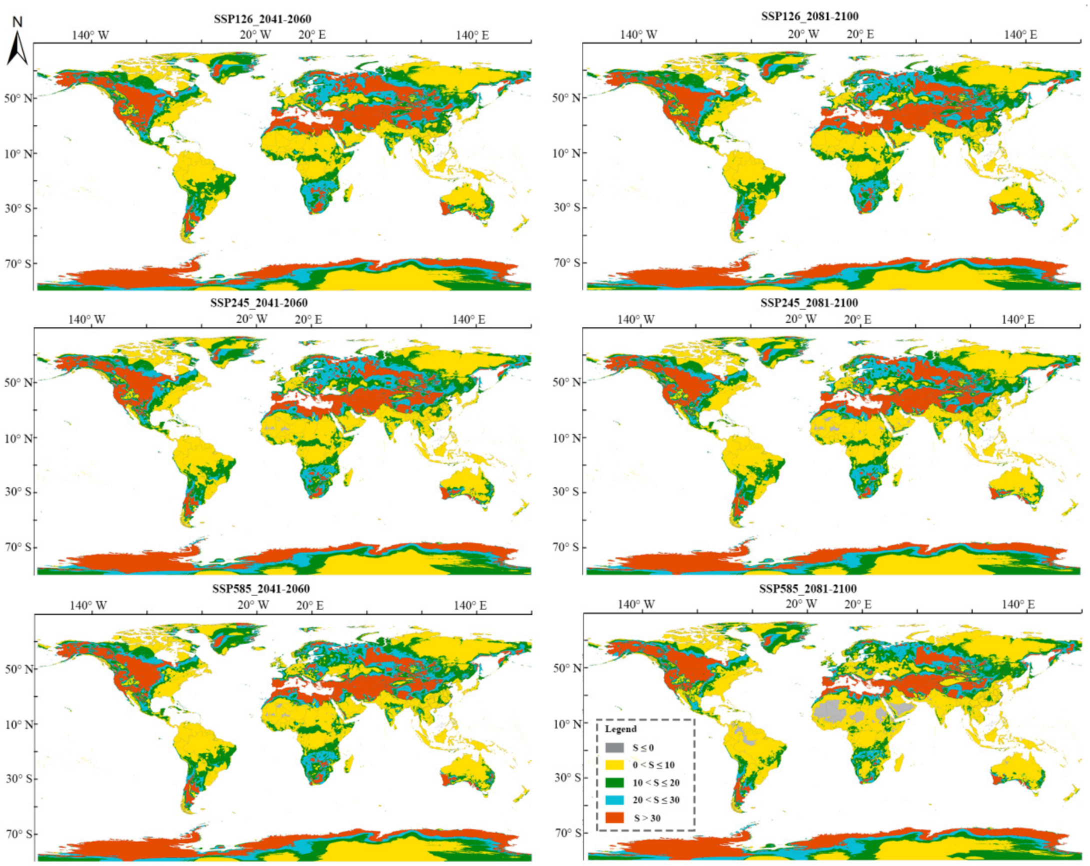

Figure S4. Multivariate environmental similarity surface (MESS) for VMF under SSP126,

SSP245 and SSP585 in 2041-2060 and 2081-2100.

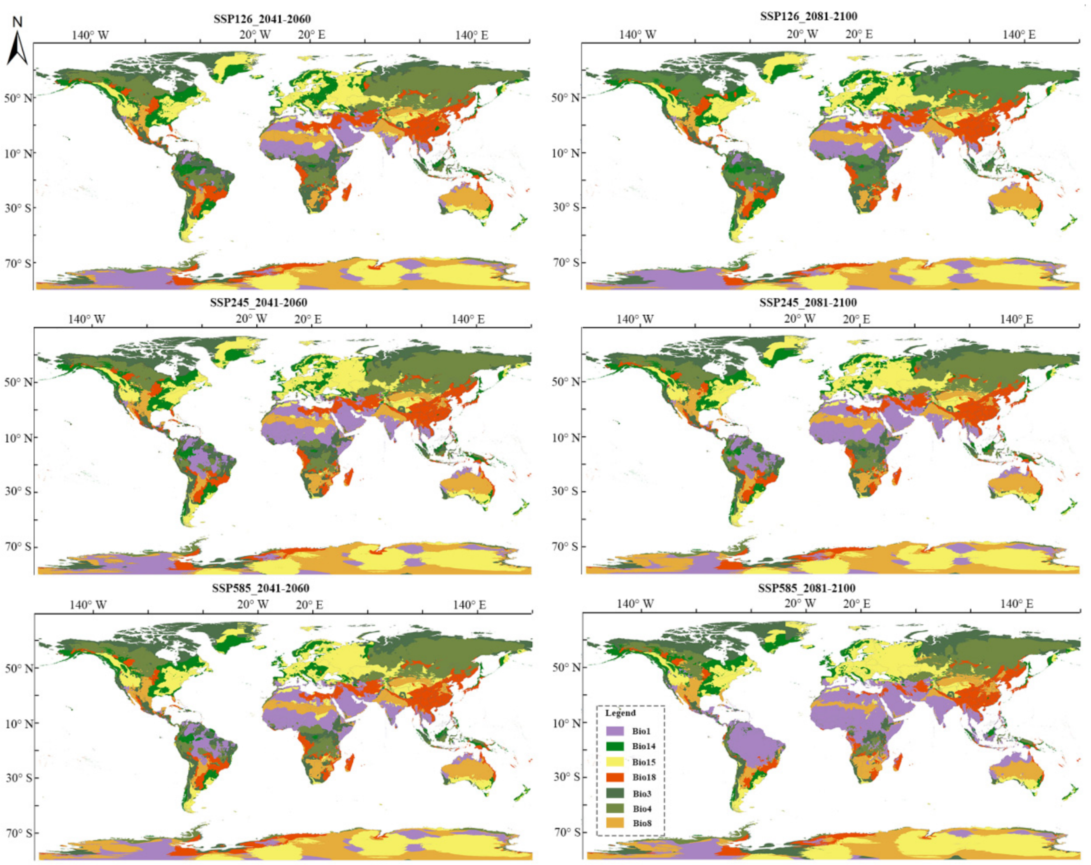

Figure S5. Most dissimilar (MoD) for VMF SSP126, SSP245 and SSP585 in 2041-2060 and 2081-2100.

Tables S1. Environmental variables and their descriptionsused for MaxEnt modeling in VMF

Variable	Description	Percent contribution (%)	Permutation Importance (%)
Bio1	Annual mean temperature [°C]	1	1
Bio2	Mean diurnal temperature range (mean of monthly (max temp−min temp)) [°C]	1.1	2.1
Bio3	Isothermality (bio2/bio7)(×100) [/]	7.4	18.6
Bio4	Temperature seasonality (standard deviation×100) [/]	5.8	1.1
Bio5	Max temperature of warmest Month [°C]	0.2	0.6
Bio6	Min temperature of coldest month [°C]	1.7	19.6
Bio7	Temperature annual range [°C]	4	1.1

Bio8	Mean temperature of wettest quarter [℃]	0.8	0.8
Bio9	Mean temperature of driest quarter [℃]	0.1	1.2
Bio10	Mean temperature of warmest quarter [℃]	0.1	0
Bio11	Mean temperature of coldest quarter [℃]	0.7	0.5
Bio12	Annual precipitation [mm]	0.7	1.1
Bio13	Precipitation of wettest month [mm]	0.3	1.7
Bio14	Precipitation of driest month [mm]	1.1	3
Bio15	Precipitation seasonality (coefficient of variation) [/]	10.8	18.8
Bio16	Precipitation of wettest quarter [mm]	5	1
Bio17	Precipitation of driest quarter [mm]	0.3	0.2
Bio18	Precipitation of warmest quarter [mm]	58.7	27.3
Bio19	Precipitation of coldest quarter [mm]	0.1	0.2

Tables S2. The Pearson correlation coefficient between each environmental factor for VMF

	Bio1	Bio2	Bio3	Bio4	Bio5	Bio6	Bio7	Bio8	Bio9	Bio10	Bio11	Bio12	Bio13	Bio14	Bio15	Bio16	Bio17	Bio18	Bio19
Bio1	1																		
Bio2	.746**	1																	
Bio3	-.463**	-.919**	1																
Bio4	0.001	-0.053	0.021	1															
Bio5	0.069	.541**	-.721**	.593**	1														
Bio6	-0.084	-.711**	.904**	0.14	-.714**	1													
Bio7	-0.033	0.073	-0.121	.837**	.672**	-0.099	1												
Bio8	.254**	.623**	-.742**	.592**	.951**	-.655**	.641**	1											
Bio9	-.193**	-.149*	0.065	.958**	.615**	0.077	.892**	.576**	1										
Bio10	.280**	.688**	-.805**	.552**	.973**	-.716**	.633**	.970**	.540**	1									
Bio11	-0.132	.190**	-.338**	.169*	.479**	-.443**	.295**	.482**	.269**	.445**	1								
Bio12	0.019	.234**	-.318**	0.089	.358**	-.362**	.204**	.403**	0.135	.350**	.913**	1							
Bio13	-.583**	-.625**	.491**	.321**	-0.03	.316**	0.134	-0.081	.425**	-.155*	0.067	-.160*	1						
Bio14	.457**	.407**	-.297**	-0.109	0.054	-.161*	0.007	.157*	-.189*	0.141	.323**	.629**	-.676**	1					
Bio15	0.019	.266**	-.357**	0.077	.388**	-.411**	.220**	.429**	0.139	.384**	.947**	.988**	-.150*	.589**	1				

Bio16	-.508**	-.555**	.415**	.369**	0.037	.275**	0.131	0.013	.431**	-0.086	.231**	0.085	.899**	-.452**	0.069	1			
Bio17	-0.071	.195**	-.267**	-.160*	.221**	-.412**	0.139	.219**	-0.019	.215**	.848**	.850**	-.167*	.469**	.881**	-0.063	1		
Bio18	-.448**	-.499**	.360**	.361**	0.059	.241**	0.117	0.059	.407**	-0.055	.288**	.168*	.831**	-.359**	.144*	.969**	-0.024	1	
Bio19	0.138	.442**	-.555**	.782**	.948**	-.485**	.824**	.934**	.792**	.940**	.443**	.328**	0.055	0.053	.354**	0.114	.172*	0.127	1

Note: **. $p < 0.01$; *. $p < 0.05$.

Table S3. Predicted suitable areas for VMF under the current and future climatic conditions

SSP		Predicted Area (10^4 km)			Comparison with Current Distribution (%)		
		High Suitable	Medium Suitable	Low Suitable	High Suitable	Medium Suitable	Low Suitable
	Current	260.59	169.15	370.46	-	-	-
SSP126	2041 to 2060s	263.90	188.83	366.95	1.27	11.64	-0.95
	2061 to 2080s	279.26	176.03	366.26	7.17	4.07	-1.14
	2081 to 2100s	260.43	180.36	376.65	-0.06	6.63	1.67
SSP245	2041 to 2060s	242..02	177.66	377.25	-7.13	5.04	1.83
	2061 to 2080s	276.36	172.06	372.08	6.05	1.72	0.44
	2081 to 2100s	249.21	196.29	361.20	-4.37	16.05	-2.50
SSP585	2041 to 2060s	254.67	186.00	372.08	-2.27	9.96	0.44
	2061 to 2080s	268.14	173.71	378.55	2.90	2.70	2.18
	2081 to 2100s	251.10	173.81	369.58	-3.64	2.75	-0.24

Note: SSP126: "Green Road" scenario with low GHG emissions, sustainable development, and minimal climate mitigation/adaptation challenges; SSP245: "Middle of the Road" scenario with moderate GHG emissions, typical development, and medium climate mitigation/adaptation

challenges; SSP585: Fossil-fueled scenario with high GHG emissions, energy-intensive growth, and major climate mitigation challenges.
